# Scale development and utilization of universal PCR-based and high-throughput KASP markers specific for chromosome arms of rye (*Secale cereale* L.)

**DOI:** 10.1186/s12864-020-6624-y

**Published:** 2020-03-04

**Authors:** Guohao Han, Shiyu Liu, Yuli Jin, Mengshu Jia, Pengtao Ma, Hong Liu, Jing Wang, Diaoguo An

**Affiliations:** 1 Center for Agricultural Resources Research, Institute of Genetics and Developmental Biology, Chinese Academy of Sciences, Shijiazhuang, 050021 Hebei China; 2 University of Chinese Academy of Sciences, Beijing, 100049 China; 3 School of Life Sciences, Yantai University, Yantai, 264005 Shandong China; 4 The Innovative Academy of Seed Design, Chinese Academy of Sciences, Beijing, China

**Keywords:** Rye, Chromosome-specific marker, KASP marker, MAS, Wheat

## Abstract

**Background:**

Rye (*Secale cereale* L., 2n = 2x = 14, RR), a relative of common wheat, is a large gene resource pool for wheat improvement. Accurate and convenient identification of the rye chromatin in wheat background will facilitate the transfer and utilization of elite genes derived from rye in wheat breeding.

**Results:**

In the present study, five rye cultivars including Imperial, German White, Jingzhouheimai, Baili and Guyuan were sequenced by specific-locus amplified fragment sequencing (SLAF-seq) to develop large-scale rye-specific markers. Based on SLAF-seq and bioinformatics analyses, a total of 404 universal PCR-based and a whole set of Kompetitive allele-specific PCR (KASP) markers specific for the 14 individual rye chromosome arms were developed and validated. Additionally, two KASP markers specific for 1RS and 2RL were successfully applied in the detection of 1RS translocations in a natural population and 2RL chromosome arms in wheat-rye derived progenies that conferred adult resistance to powdery mildew.

**Conclusion:**

The 404 PCR-based markers and 14 KASP markers specific for the 14 individual rye chromosome arms developed in this study can enrich the marker densities for gene mapping and accelerate the utilization of rye-derived genes in wheat improvement. Especially, the KASP markers achieved high-throughput and accurate detection of rye chromatin in wheat background, thus can be efficiently used in marker-assisted selection (MAS). Besides, the strategy of rye-specific PCR-based markers converting into KASP markers was high-efficient and low-cost, which will facilitate the tracing of alien genes, and can also be referred for other wheat relatives.

## Background

Common wheat (*Triticum aestivum* L.) is a major grain crop worldwide. With the expanding global population to nine billion by 2050, wheat production is facing a challenge of about 70% growth to meet the demands in the future [1]. However, wheat breeding mainly focused on crossing between cultivars for a long time, which resulted in more homogeneous genetic backgrounds and narrowed genetic diversity in wheat breeding [2]. The wheat relatives have significant genetic diversity and abundant valuable genes, therefore can play an important role in wheat improvement [3]. To date, many elite alien genes and desirable traits have been transferred into common wheat through hybridization and chromosome engineering, such as disease resistance, superior yield-related traits, salt and drought tolerance [4–7].

Rye (*Secale cereale* L., 2n = 2x = 14, RR), a naturally cross-pollinated relative of common wheat, can be used as a huge gene donor for wheat improvement. For example, the wheat-rye T1RS·1BL translocation derived from rye cultivar Petkus carries the powdery mildew resistance gene *Pm8*, stripe rust resistance gene *Yr9*, leaf rust gene *Lr26* and stem rust resistance gene *Sr31* [8, 9], along with superior agronomic traits and abiotic stress tolerance [10, 11]. Therefore, the T1RS·1BL translocation has been widely used worldwide and was regarded as a particular notable success in crop improvement of alien chromosomes [12, 13]. Apart from the 1RS, some other chromosomes of rye carrying resistance genes have also been transferred into common wheat in forms of translocations, including *Pm7* and *Lr25* on 2RL from rye cultivar Rosen [14, 15], *Lr45* on 2RL from rye cultivar Petkus [16], *Sr59* on 2RL from triticale VTB28041 [17], *Sr27* on 3RS from rye cultivar Imperial [18], *Pm56* on 6RS from rye cultivar Qinling [19] and *Pm20* on 6RL from rye cultivar Prolific [14]. In addition, increasing rye-derived genes have been used in wheat improvement in recent years. For example, Schneider et al. [12] demonstrated that chromosome 1R, 4R, and 6R from rye cultivar Perennial could increase arabinoxylan and protein content after transferring into wheat background. A wheat-rye 4R addition line increased kernel number per spike after the 4R transferring into wheat [20].

After transferring alien chromatin into common wheat, it is important to develop rapid, accurate and convenient methods to trace them. Genomic in situ hybridization (GISH) and fluorescence in situ hybridization (FISH), especially multicolor FISH (mc-FISH), are widely-used detection methods owing to intuitive and accurate specialty [21, 22]. Meanwhile, molecular markers specific for alien chromosomes are also powerful for detecting alien chromatin in wheat background [23]. Therefore, a high-efficient strategy for developing specific molecular markers played a key role in the utilization of alien genes. Despite a series of rye-specific markers have been reported [24–28], it is still in a large demand when applied in high-resolution mapping, population genetic studies and marker assisted selection (MAS). In addition, as a cross-pollinated crop, rye contains significant genetic heterogeneity within and among cultivars [29, 30]. This will limit the universality of the specific markers in different rye genetic backgrounds. Therefore, it is necessary to develop a large number of universal, stable and easily performed markers for construction of high-density map of rye, detection of rye chromatin in wheat backgrounds and MAS.

With rapid advancement of next-generation sequencing technology (NGS) and low-cost genome sequencing, an increasing number of single nucleotide polymorphism (SNP) markers were developed attributing to their high stability, high resolution and low cost, and therefore are suitable for large-scale genotyping [31–33]. On this basis, Kompetitive allele-specific PCR (KASP) assay method, as one of current SNP assay platforms, has been successfully used to identify alien chromatin in wheat background, which can accelerate the tracking of alien fragments and improved the efficiency of MAS [34]. However, many wheat relatives are short of high-quality whole-genome sequence, which has limited the development of KASP markers for the detection of alien segments on a large scale.

In this study, an efficient NGS method specific-locus amplified fragment sequencing (SLAF-seq) and bioinformatics analyses were combined to develop a large number of universal PCR-based markers distributed on all the chromosome arms of rye. Then, these markers were analyzed to generate a set of KASP markers specific for each arm of the rye chromosome. Furthermore, these KASP markers were validated and applied in MAS on scale. The strategy for development of KASP markers adopted in this study can be a good reference for other wheat relatives.

## Results

### Development and verification of universal PCR-based markers

Based on the results of high-throughput sequencing method SLAF-seq for the five rye cultivars, including Imperial, German White, Jingzhouheimai, Baili and Guyuan, a total of 653,144 SLAFs were acquired. The average Q30 ratio was 86.79%, indicating that the data has high quality. By sequence alignment between the five rye cultivars and Chinese Spring [35], 3871 sequences with homology less than 50% of wheat genome were obtained and considered as the conserved and rye-specific sequences. A total of 1546 SLAFs were randomly selected to design rye-specific PCR-based primers. Among them, 667 primers which amplified specific bands in all the five rye cultivars plus KingII but not in the wheat cultivar Holdfast were regarded as the universal rye-specific markers.

**Fig. 1:**
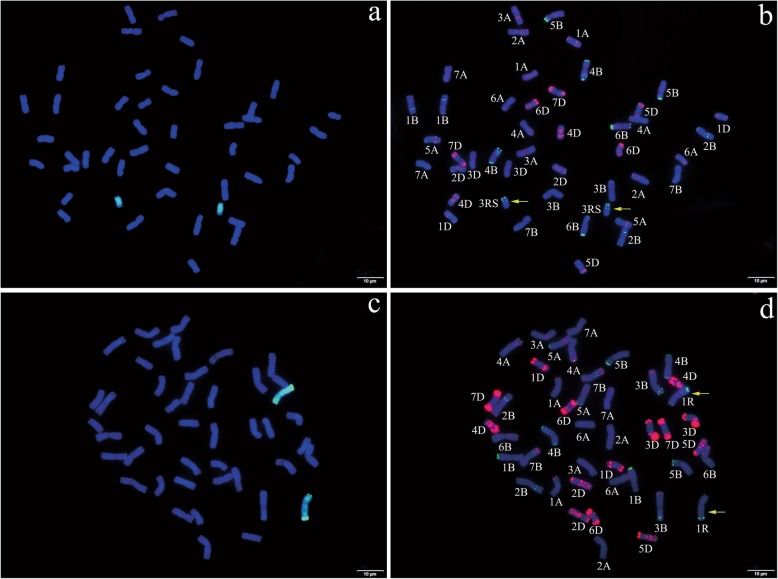
Genomic in situ hybridization (GISH) and non-denaturing fluorescence in situ hybridization (ND-FISH) analyses of 3RS ditelosomic addition line of ‘Holdfast-KingII’ and 1R disomic addition line of ‘Chinese Spring-Imperial’. For GISH, the rye genomic DNA (green) was used as a probe and Chinese Spring DNA as a blocker. Chromosomes were counterstained with DAPI (blue). **a** GISH analysis of 3RS ditelosomic addition line of ‘Holdfast-KingII’. **b** ND-FISH analysis of the same metaphase cell after GISH analysis (**a**) with Oligo-pSc119.2–1 (green) and Oligo-pTa535–2 (red). **c** GISH analysis of 1R disomic addition line of ‘Chinese Spring-Imperial’. **d** ND-FISH analysis of same metaphase cell with after GISH analysis (**c**) with Oligo-pSc119.2–1 (green) and Oligo-pAs1–1 (red). The bar represents 10 μm and the arrows represent rye chromosomes or chromosome arms

Then, a complete set of wheat-rye disomic and ditelosomic addition lines of ‘Holdfast-KingII’ and a set of wheat-rye disomic addition lines of ‘Chinese Spring-Imperial’ were used for the location and verification of these 667 markers. All of the wheat-rye addition lines were clearly identified to contain the two corresponding rye chromosome arms or chromosomes by GISH and non-denaturing FISH (ND-FISH) analyses, such as the 3RS ditelosomic addition line of ‘Holdfast-KingII’ identified by the probes of Oligo-pSc119.2–1 and Oligo-pTa535–2 (Fig. 1a and b) and the 1R disomic addition line of ‘Chinese Spring-Imperial’ identified by the probes of Oligo-pSc119.2–1 and Oligo-pAs1–1 (Fig. 1c and d).

**Fig. 2:**
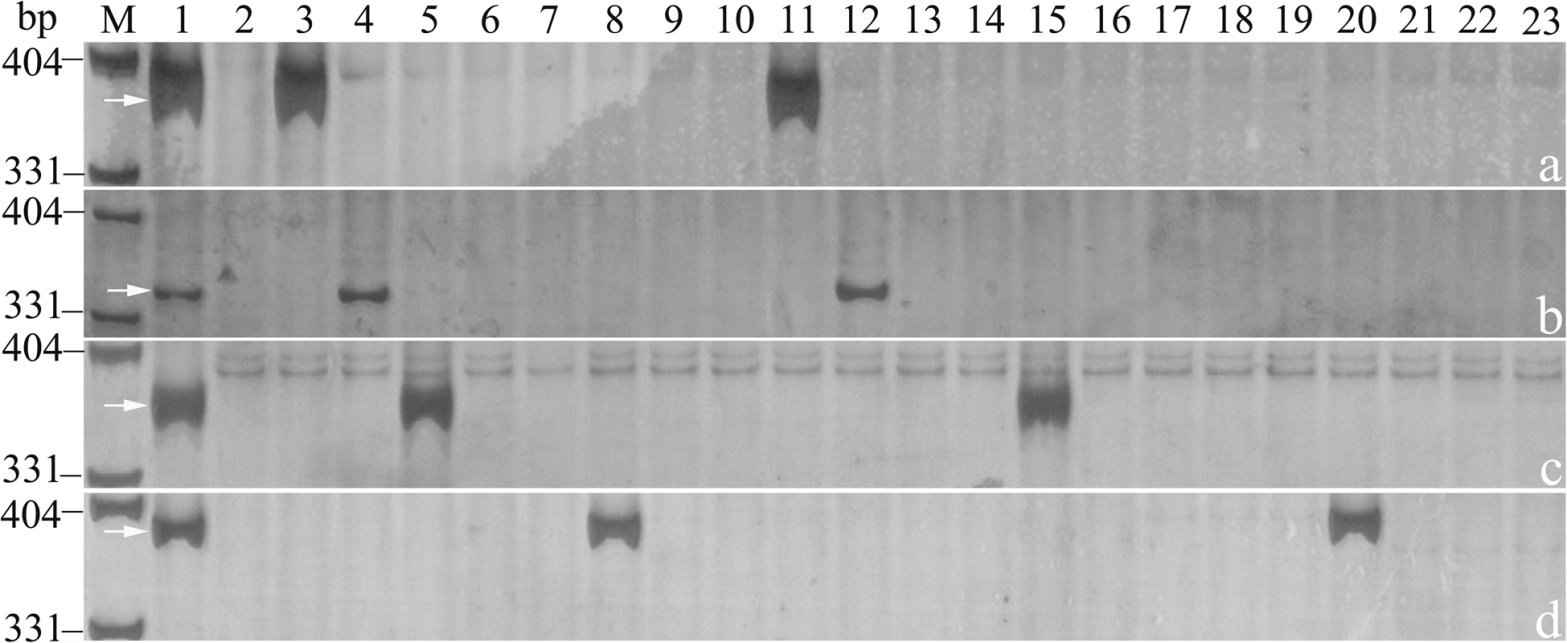
PCR amplification for location of 1RS-specific marker SW5282, 2RL-specific marker SW252224, 3RS-specific marker SW28002 and 6RL-specific marker SW26615 (**a**-**d**) on corresponding rye chromosome arms, respectively. The arrows represent targeted bands. M: pUC19/*Msp*I, 1: KingII, 2: Holdfast, 3–9: 1R-7R disomic addition lines of ‘Holdfast-KingII’, 10–23: 1RL, 1RS-7RL and 7RS ditelosomic addition lines of ‘Holdfast-KingII’

**Fig. 3:**
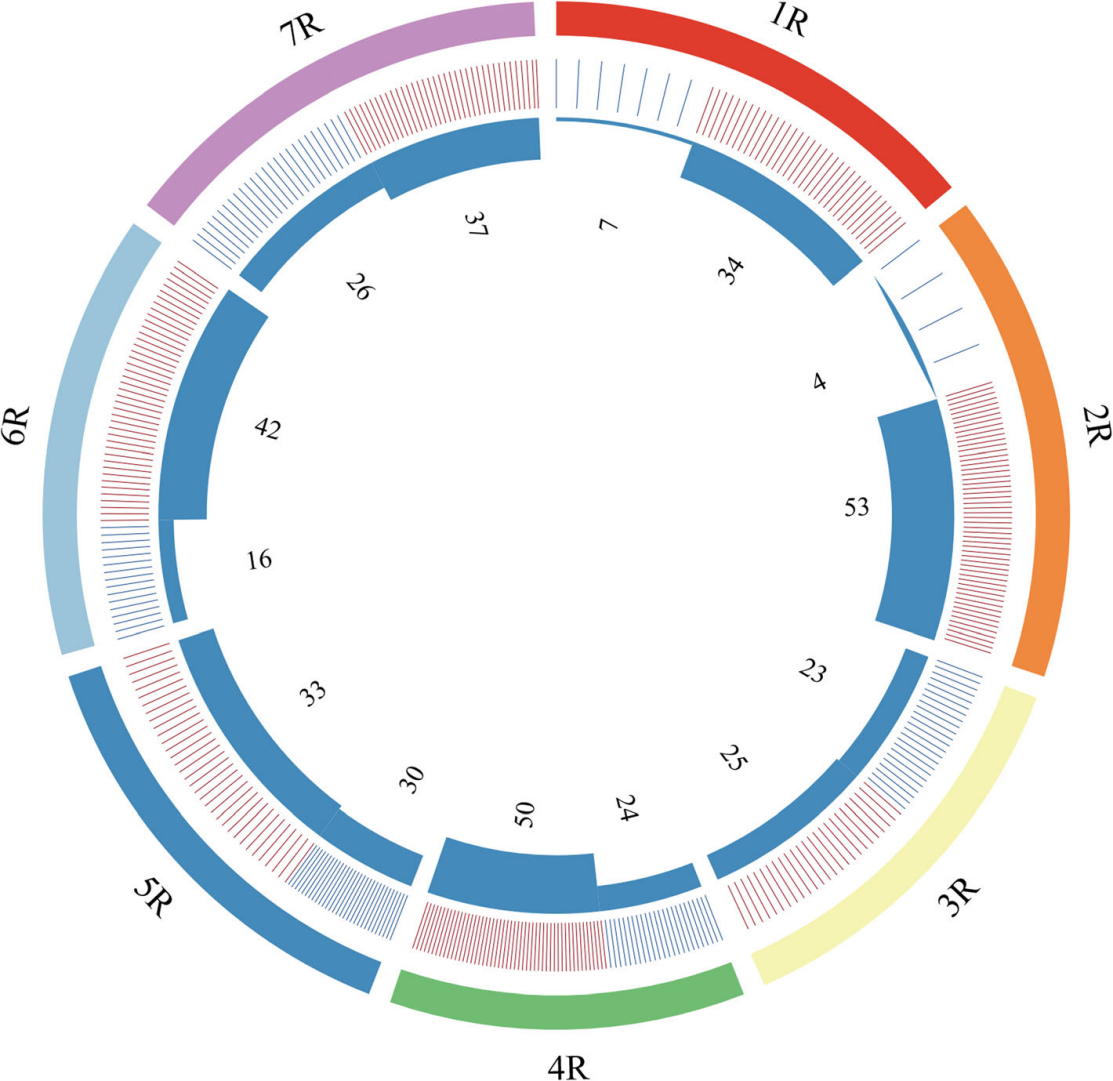
Frequency and number of the markers assigned to the individual rye chromosome arms. The outer track is separated to seven circular tracks showing the seven chromosomes 1R to 7R of rye. The second track is formed by blue bars and red bars which indicate markers assigned to short arm and long arm, respectively. The density of the bars illustrates the frequency of the markers assigned to the individual rye chromosome arms. The inner blue blocks present the number of the markers located on the individual rye chromosome arms

The markers which amplified specific bands in rye cultivar KingII, and only one of wheat-rye disomic addition lines of ‘Holdfast-KingII’ but not in others were regarded as rye chromosome-specific markers. As a result, 418 markers were located to specific chromosomes of rye, including 43, 58, 49, 74, 64, 62 and 68 markers on rye chromosome 1R, 2R, 3R, 4R, 5R, 6R and 7R, respectively. Subsequently, the assignments of theses markers to individual chromosome arms were determined using KingII, Holdfast, and a set of wheat-rye disomic addition lines and ditelosomic addition lines of ‘Holdfast-KingII’. Consequently, 404 markers that amplified specific bands in KingII, one of wheat-rye disomic addition lines and only one of corresponding ditelosomic addition lines of ‘Holdfast-KingII’ but not in others were obtained. Examples of amplification bands from four specific markers, SW5282 for 1RS, SW252224 for 2RL, SW28002 for 3RS and SW26615 for 6RL, are showed in Fig. 2. Among the 404 rye chromosome arm-specific markers, 7, 34, 4, 53, 23, 25, 24, 50, 30, 33, 16, 42, 26, 37 markers were successively assigned to 1RS, 1RL, 2RS, 2RL, 3RS, 3RL, 4RS, 4RL, 5RS, 5RL, 6RS, 6RL, 7RS and 7RL arms of rye chromosome, respectively (Fig. 3). Each of the markers was referred to individual SLAF, except for the seven and four markers assigned to 1RS and 2RS, respectively. Only two markers were assigned to each of these two arms initially, but in order to increase the markers available, a total of seven and four markers located on 1RS and 2RS were redesigned based on the sequence of original scaffold of *S. cereale* L. Lo7 [36] which it belongs to. The primer sequences of these markers are presented in Additional file 1: Table S1.

**Fig. 4:**
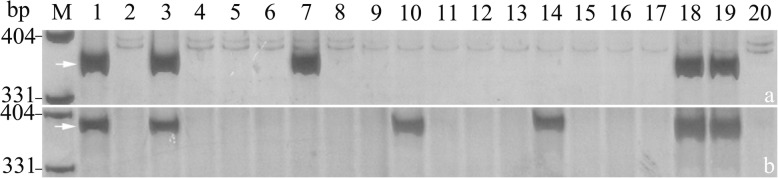
PCR amplification for verification of 3RS-specific marker SW28002 (**a**) and 6RL-specific marker SW26615 (**b**). The arrows represent targeted bands. M: pUC19/*Msp*I, 1: KingII, 2: Holdfast, 3: Imperial, 4: Chinese Spring, 5–11: 1R-7R disomic addition lines of ‘Chinese Spring-Imperial’, 12: Wheat-rye 4R disomic addition line WR35, 13: T4BL·4RL and T7AS·4RS translocation line WR41, 14: 6R disomic addition line WR49, 15: 2RL ditelosomic addition line WR56, 16: 2R (2D) disomic substitution line WR91, 17: T1RS·1BL translocation line Lovrin10, 18: octoploid triticale line 09R1–38, 19: octoploid triticale line 09R1–100, 20: Shixin633

Then, the 404 markers were valuated their specificity, stability and universality with KingII, Holdfast, Imperial, Chinese Spring, a set of wheat-rye disomic addition lines of ‘Chinese Spring-Imperial’, wheat-rye lines WR35, WR41, WR49, WR56, and WR91 which involved different rye chromosomes or chromosome arms, T1RS·1BL translocation line Lovrin10, two octoploid triticale lines 09R1–38 and 09R1–100, and wheat cultivar Shixin633. For instance, the 3RS-specific marker SW28002 and 6RL-specific marker SW26615 were successfully validated to amplify the same specific bands in Imperial, corresponding addition line of ‘Chinese Spring-Imperial’ and two octoploid triticale lines 09R1–38 and 09R1–100 as in KingII. SW26615 also amplified the specific bands in the wheat-rye 6R addition line WR49 (Fig. 4). All of the markers could amplify specific bands in the corresponding wheat-rye addition lines of ‘Chinese Spring-Imperial’ and materials. Thus, the specificity, stability and universality of the 404 universal rye chromosome arm-specific PCR-based markers were finally confirmed.

### Development and validation of rye specific KASP markers

In order to achieve higher efficiency of these markers in MAS, a whole set of KASP markers specific for the 14 individual rye chromosome arms were developed and validated. Firstly, 14 PCR-based markers have already been assigned to the 14 rye chromosome arms were randomly selected to convert to KASP markers. According to the results of sequence alignment, each of the 14 original SLAF sequences could compared to a highly homologous scaffold of *S. cereale* L. Lo7 [36], indicating the specificity of these SLAFs in rye genome. Subsequently, a large amount of targeted sequences with only one unique SNP between rye and wheat genome [35], derived from the 14 SLAFs or the expanded scaffold sequences of *S. cereale* L. Lo7 [36], respectively, were obtained via SNP calling analyses. KASP markers were designed based on the selected targeted sequences with favorable primer quality. These markers were validated using the following cultivars or lines: 12 rye cultivars including KingII, Imperial, German White, Jingzhouheimai, Baili, Guyuan, CIse 1, CIse 12, CIse14, CIse17, CIse53 and CIse54, a complete set of wheat-rye disomic and ditelosomic addition lines of ‘Holdfast-KingII’, a set of disomic addition lines of ‘Chinese Spring-Imperial’, and 11 wheat cultivars including Holdfast, Chinese Spring, Shixin633, Shixin733, Shixin828, Gao8901, Jishi02–1, Shimai15, Kenong199, Heng5471 and Jimai22. Consequently, 14 KASP markers specific for the individual 14 rye chromosome arms were successfully developed (Table 1).

**Table 1:** Primer sequences of Kompetitive allele-specific PCR (KASP) markers specific for 14 rye chromosome arms

KASP marker	Location	Primer sequences (5′-3′)
SWK5282 -F	1RS	gaaggtgaccaagttcatgctGAGCTGATTTCCATGTA
SWK5282 -H		gaaggtcggagtcaacggattGAGCTGATTTCCATGTC
SWK5282 -C		TACCAAGTCCTGAACCA
SWK23822-F	1RL	gaaggtgaccaagttcatgctTATGGGAATTTATGGCCGCA
SWK23822-H		gaaggtcggagtcaacggattTATGGGAATTTATGGCCGCG
SWK23822-C		CCCGGAAAAGCTCCTTTT
SWK621-F	2RS	gaaggtgaccaagttcatgctGAGGAAGCTCCATCAATCTG
SWK621-H		gaaggtcggagtcaacggattGAGGAAGCTCCATCAATCTT
SWK621-C		CACCGAATCAATCATGCAAC
SWK252224-F	2RL	gaaggtgaccaagttcatgctTCAACACCAAGAGAAGGGAAC
SWK252224-H		gaaggtcggagtcaacggattTCAACACCAAGAGAAGGGAAA
SWK252224-C		CAGATGCATGTAGGTAGCGC
SWK28002-F	3RS	gaaggtgaccaagttcatgctCGGACAATGCACGATCGA
SWK28002-H		gaaggtcggagtcaacggattCGGACAATGCACGATCGG
SWK28002-C		CGCACGCACATCAACACG
SWK15063-F	3RL	gaaggtgaccaagttcatgctCGAAAGTATGGGCTGCATTTT
SWK15063-H		gaaggtcggagtcaacggattCGAAAGTATGGGCTGCATTTC
SWK15063-C		CCGACCCGTTCAGCCATT
SWK30487-F	4RS	gaaggtgaccaagttcatgctGGGTCGAGGTAGGTGAGG
SWK30487-H		gaaggtcggagtcaacggattGGGTCGAGGTAGGTGAGC
SWK30487-C		GCTGACGGCACAATCAAC
SWK61253-F	4RL	gaaggtgaccaagttcatgctTGAAGTACTCAGCATTCAGC
SWK61253-H		gaaggtcggagtcaacggattTGAAGTACTCAGCATTCAGT
SWK61253-C		GTTCTCTTGTTCACACTCCAGT
SWK190654-F	5RS	gaaggtgaccaagttcatgctAGGCCAAGAGAAGAGTGAAGAC
SWK190654-H		gaaggtcggagtcaacggattAGGCCAAGAGAAGAGTGAAGAT
SWK190654-C		TAACTACCGGCTGCCCTTTT
SWK37355-F	5RL	gaaggtgaccaagttcatgctTTCTGGTCCTAACGCTGAA
SWK37355-H		gaaggtcggagtcaacggattTTCTGGTCCTAACGCTGAG
SWK37355-C		CTGCATGCAATTCAAGACAGA
SWK38534-F	6RS	gaaggtgaccaagttcatgctTGAATCTCAACCATGCCCTT
SWK38534-H		gaaggtcggagtcaacggattTGAATCTCAACCATGCCCTC
SWK38534-C		CCTTGACTGTGTGGCCGATT
SWK26615-F	6RL	gaaggtgaccaagttcatgctCACTTTAACTTGGCGTTGGAG
SWK26615-H		gaaggtcggagtcaacggattCACTTTAACTTGGCGTTGGAC
SWK26615-C		CTGAACTGGCCATTTGCA
SWK31799-F	7RS	gaaggtgaccaagttcatgctACCTGAATATTGGGCGCA
SWK31799-H		gaaggtcggagtcaacggattACCTGAATATTGGGCGCC
SWK31799-C		TCACAGATCAACCTAGCCTCC
SWK13002-F	7RL	gaaggtgaccaagttcatgctATGCTTCTGCTGGTCTCTT
SWK13002-H		gaaggtcggagtcaacggattATGCTTCTGCTGGTCTCTC
SWK13002-C		CCAATACAGGAGTAGATCGAC

**Fig. 5:**
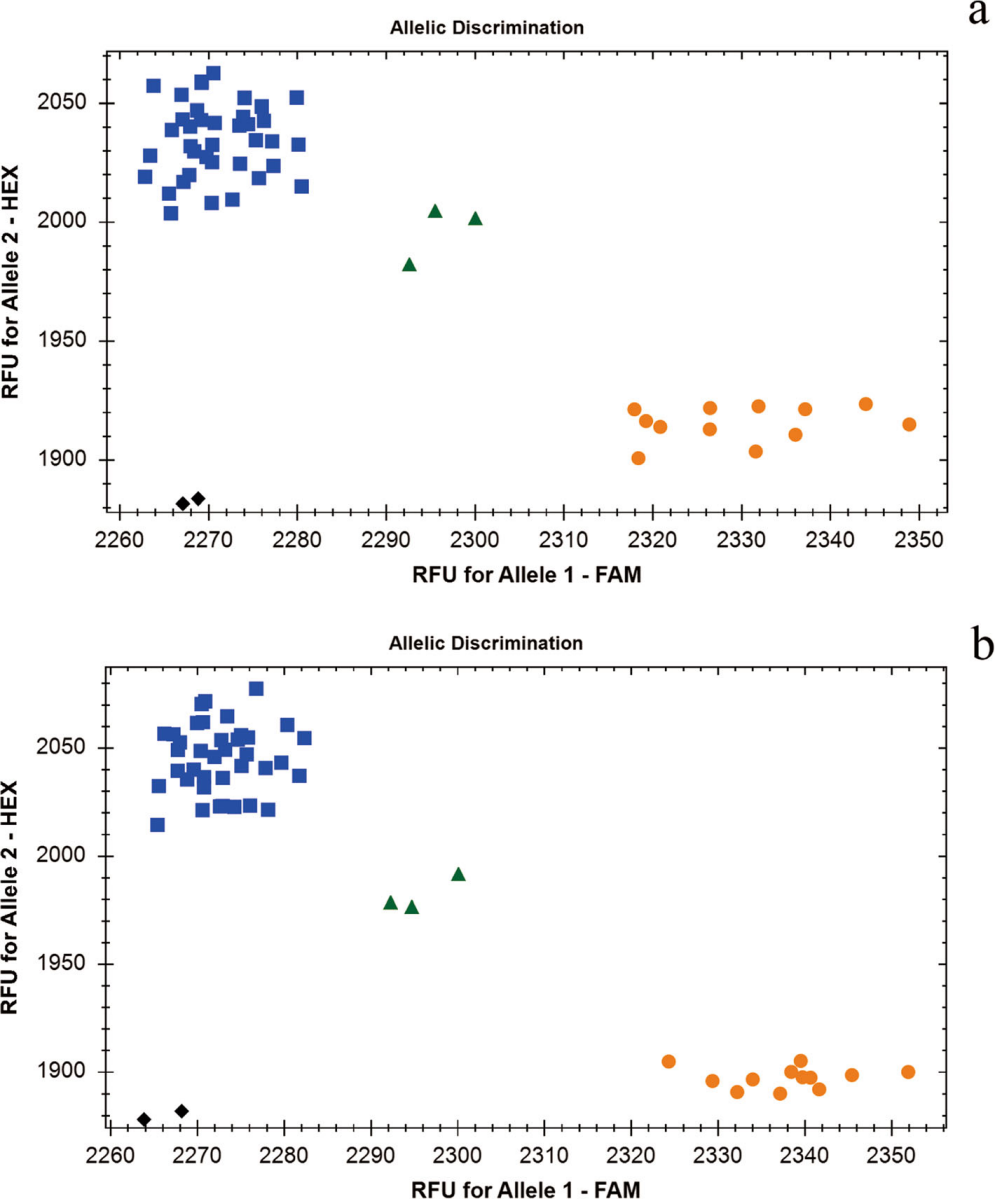
Genotyping results of 3RS-specific Kompetitive allele-specific PCR (KASP) Marker SWK28002 (**a**) and 6RL-specific KASP Marker SWK26615 (**b**). Orange rotund shapes that represent homozygous rye-derived specific SNP ‘Allele1/Allele1’ indicate 12 rye cultivars including KingII, German White, Imperial, Jingzhouheimai, Baili, Guyuan, CIse 1, CIse 12, CIse14, CIse17, CIse53 and CIse54; blue square shapes that represent homozygous wheat-derived specific SNP ‘Allele2/Allele2’ indicate two sets of disomic addition lines of ‘Holdfast-KingII’ and ‘Chinese Spring-Imperial’ without 3R/6R disomic addition lines, a set of ditelosomic addition lines of ‘Holdfast-KingII’ without 3RS/6RL ditelosomic addition line, and 11 wheat materials including Holdfast, Chinese Spring, Shixin633, Shixin733, Shixin828, Gao8901, Jishi02–1, Shimai15, Kenong199, Heng5471 and Jimai22; and green triangle shapes that represent heterozygous rye- and wheat- derived SNP ‘Allele1/Allele2’ indicate 3R/6R disomic addition line and 3RS/6RL ditelosomic addition line of ‘Holdfast-KingII’ and 3R/6R disomic addition line of ‘Chinese Spring-Imperial’. Black diamond shapes indicate no template control

The set of rye chromosome arm-specific KASP markers developed in this study were co-dominant to clearly distinguish three genotypes: two homozygous alleles indicated rye-derived SNPs or wheat-derived SNPs, and heterozygous alleles indicated presence of both wheat-derived and rye-derived SNPs. The genotyping results of 3RS-specific KASP marker SWK28002 and 6RL-specific KASP marker SWK26615 are distinctly shown in Fig. 5.

### Application of rye specific KASP markers

**Fig. 6:**
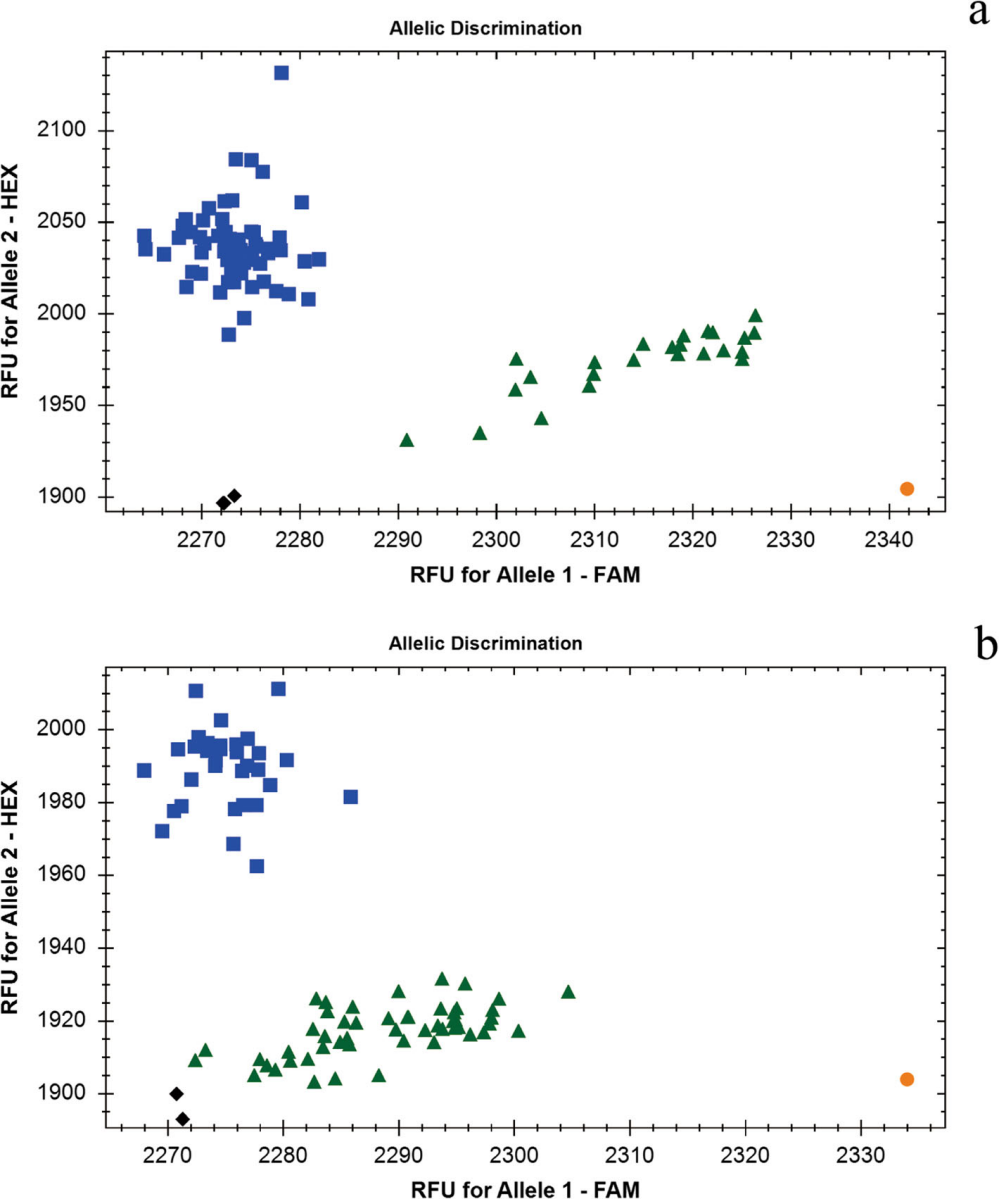
Application of 1RS-specific Kompetitive allele-specific PCR (KASP) marker SWK5282 and 2RL-specific KASP marker SWK252224. **a** Using the 1RS-specific KASP marker SWK5282, the partial genotyping results of 1RS translocation lines in 161 wheat cultivars/lines were displayed. Orange rotund shape represents the homozygous 1RS special SNP (‘Allele1/Allele1’) indicating rye Imperial, blue square shapes represent the homozygous wheat special SNP (‘Allele2/Allele2’) indicating Chinese Spring and the wheat culitivars/lines without 1RS translocations, and green triangle shapes represent the presence of both Allele1 and Allele2 indicating the wheat cultivars/lines containing 1RS translocations. Black diamond shapes indicate no template control. **b** Using the 2RL-specific KASP marker SWK252224, 2RL chromosomes in 81 F_2_ individuals of WR91 and Shixin633 were identified. Orange rotund shape represents the homozygous 2RL special SNP (‘Allele1/Allele1’) indicating rye Imperial, blue square shapes represent the homozygous wheat special SNP (‘Allele2/Allele2’) indicating Chinese Spring and F_2_ individuals without 2RL chromosome, and green triangle shapes represent the presence of both Allele1 and Allele2 indicating F_2_ individuals containing 2RL chromosome. Black diamond shapes indicate no template control

**Fig. 7:**
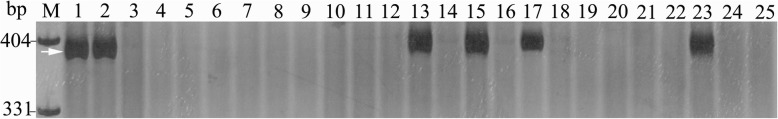
PCR amplification of 1RS-specific PCR-based marker SW5282 for the detection of 1RS translocations in part of 161 wheat cultivars/lines. M: pUC19/*Msp*I, 1: Imperial, 2: Lumai5, 3: Luami13, 4: Lumai23, 5: Liangxing66, 6: Liangxing99, 7: Jimai19, 8: Jimai20, 9: Jimai22, 10: Jinan17, 11: Tainong18, 12: Tainong19, 13: Jinan16, 14: Shannong17, 15: Luyuan502, 16: Shannong21, 17: DH5197, 18: Wennong14, 19: Lumai21, 20: Yannong19, 21: Yannong21, 22: Yannong23, 23: Weimai8, 24: Yannong24, 25: Jimai21

Using the 1RS-specific KASP marker SWK5282, the detection of 1RS translocations in a natural population with 161 wheat cultivars/lines was clearly and intuitively displayed (Fig. 6a). The distribution of 1RS translocations in 161 wheat cultivars/lines were also confirmed by the corresponding PCR-based marker SW5282 (Fig. 7). The results indicated that 78 of 161 wheat cultivars/lines contained 1RS translocations, which were consistent with the results of PCR-based marker SW5282, also provided guidance to use 1RS translocation lines in MAS breeding for wheat breeders (Additional file 2: Table S2).

WR91 was a wheat-rye 2R (2D) substitution line which was previously confirmed to exhibit adult resistance to powdery mildew on 2RL chromosome. To further transfer the resistance into susceptible commercial cultivar, WR91 has been crossed with Shixin633 and generated F_2_ populations. The 2RL-specific KASP marker SWK252224 was used to rapidly detect the 2RL chromosomes. Among the tested 81 F_2_ individuals, 50 individuals as shown in green triangle shapes were resistant to powdery mildew at adult stage with infection types 0–2, whereas the other 31 individuals as shown in blue square shapes were susceptible with infection types 7–9 (Fig. 6b). In addition, the detection results of KASP marker SWK252224 were identical to those of the corresponding PCR-based marker SW252224. The application of the KASP marker SWK252224 will greatly facilitated the detection and transfer of 2RL chromosome into wheat background.

## Discussion

Rye is an important gene donor for disease resistance, superior agronomic traits and environment adaptation in wheat breeding. A great number of desirable wheat-rye progenies from various rye accession have been developed by distant hybridization and chromosome engineering, such as wheat-rye 6R disomic addition line WR49 carrying powdery mildew resistance derived from rye cultivar German White [37], wheat-rye 1R disomic addition line N9436B carrying powdery mildew resistance derived from rye cultivar Austrian [38], and wheat-rye T1RS·1BL translocation lines from Chinese rye cultivar Baili with resistances to stripe rust and powdery mildew and desirable agronomic traits [39]. Compared with addition lines and substitution lines, translocation lines, especially small segment translocation lines carrying desirable traits, are preferred attributing to less linkage drag. Therefore, more wheat-rye introgression lines with small segments have been induced and produced [40, 41]. In this case, development of high-density rye-specific molecular markers is necessary and urgent. Besides, rye has strong heterogeneity. Guo et al. [42] identified 300 individuals from six open-pollinated rye cultivars, while no identical karyotypes were found among these individuals. This demonstrated that some rye chromosome-specific markers are not able to identify a given chromosome across all rye cultivars. In most of the previous studies, researchers usually developed rye-specific markers basing on the obtained sequences from one kind of rye cultivar or only used one set of wheat-rye addition lines to assign them, without confirming their universality in many other rye cultivars [24, 25, 43–45]. In the present study, a total of 404 PCR-based rye chromosome-specific markers were developed based on the conserved sequence across the five rye cultivars and verified with the sixth rye cultivar KingII. Meanwhile, two sets of wheat-rye addition lines were used to assign the markers to individual chromosomes. Thus, theses markers were supposed to be more universal, enable the identification of alien chromatin of various rye cultivars, and provide powerful tools for constructing map of rye and MAS.

Previously, developing markers was low-efficient due to the lack of genome reference information of the wheat relatives. SLAF-seq is an efficient next-generation sequencing method that can rapidly obtain a large amount of high-quality enzymatic fragments [46]. Therefore, SLAF-seq can be effectively applied to development of alien chromosome specific markers [47, 48]. In the present study, 418 markers derived from 1546 SLAFs were assigned to rye chromosomes, and finally 404 markers were assigned to universal chromosome arms, with a success rate of up to 26%. In addition, the frequency of markers located on most of chromosome arms was relatively uniform, except for the fewer markers on chromosome arms 1RS and 2RS, which was probably caused by fewer distribution of the selected restriction fragments on these two arms. All these PCR-based markers were used easily to trace the rye chromosome segments in wheat background.

Recent advances in NGS promoted the construction of reference sequence assemblies and development of high-density DNA genotyping platforms for crops. Winfield et al. [49] developed high-density SNP genotyping array for hexaploid wheat and its secondary and tertiary gene pool based on exome capture and NGS, which can achieve the tracking of the introgression and subsequent fate of chromosomal segments from wheat relatives in wheat breeding. A Wheat-Relative SNP Genotyping Array has also been developed and used to identify the wild relative introgressions in a wheat background [50–53]. However, these SNP genotyping platforms are limited in practical breeding due to their inflexibility in assay design, identification and costly equipment for application, although they are high-throughput and high-efficient [54]. With the help of the published sequencing data of wheat, KASP marker has been widely used in mapping and MAS due to its advantages of high throughput, high flexibility, good genetic stability, locus specificity and low cost [31, 55]. Compared with common wheat, the lack of high-quality whole genome sequencing information of wheat relatives limits the development of alien specific KASP markers and its utilization in MAS. Tiwari et al. [56] developed KASP markers for detecting 5M^g^ segments in wheat-*Aegilops geniculate* introgression lines by combining chromosome flow-sorting and next-generation sequencing technology, while the flow-sorting technology is expensive and not yet widespread. Rahmatov et al. [17] developed three 2RL-specific KASP markers with two genotypes: lines with targeted segment displayed the rye allele, whereas the lines without targeted segments displayed null allele. Ma et al. [34] developed the co-dominant KASP markers that were able to detect *Agropyron cristatum* chromosome based on the RNA-seq data. Recently, Grewal et al. [57] developed chromosome-specific KASP genotyping assays for ten wild relative species based on the probe sequences on the Axiom® Wheat-Relative Genotyping Array. These markers that have been developed will provide powerful tools for wheat breeders worldwide.

Compared with the reported studies, the strategy of developing alien specific KASP markers in this study was low-cost and high-efficient. In this study, 14 specific markers distributed on the 14 arms were selected and finally a whole set of rye chromosomal arm-specific KASP markers were developed and valuated. Firstly, SLAF-seq was used to develop PCR-based markers. Subsequently, the original SLAF sequences of the specific markers or the expanded sequences obtained by the *S. cereale* L. Lo7 scaffolds database [36] were compared with the Chinese Spring reference sequences [35] to search the single unique SNP. Finally, the specific KASP markers were developed based on the targeted SNP. Actually, the results of sequence alignment with the data of *S. cereale* L. Lo7 scaffolds [36] have proved that the PCR-based specific markers obtained in the first step could largely ensure the sequence specificity and accuracy. For the second step, the reference genome sequencing information of the wheat relatives are not necessary if the specific SLAFs on targeted chromosomes are enough. Therefore, this strategy can be a good reference for other wheat relatives.

The whole set of 14 rye chromosome arm-specific KASP markers could clearly distinguish three genotypes for wheat, rye, and wheat-rye introgression. Meanwhile, the targeted single unique SNPs with wheat were sequenced to be conserved among five rye cultivars as well it was turned out in six other rye cultivars, indicating the universality of these KASP markers in various rye cultivars. For the detection of 1RS translocations in a natural population with 161 wheat cultivars/lines, SWK5282 was more time-saving and convenient than PCR-based marker SW5282. The result also demonstrated that this KASP marker can be applied to screen wheat-rye derived populations on large scale, unconstrained by different wheat and rye backgrounds. In addition, the introduction of 1RS arms into common wheat leads to defects in bread-making quality while improving yield [58], the detection results in this study thereby provide guidance for parent selection in wheat breeding. For the tracing of the powdery mildew resistance derived from chromosome 2RL, the co-segregation of the resistance phenotype with KASP marker SWK252224 indicated that this marker could be used in high-throughput detection of 2RL chromosome in wheat-rye derived populations, significantly accelerating the transfer of resistance genes into susceptible wheat cultivars. Recently, two 4RL-specific markers have been successfully developed and applied in MAS using this strategy in our lab [59]. Actually, many valuable alien genes were transferred into wheat mainly in forms of stable Robertsonian translocations, such as wheat-*Dasypyrum villosum* translocation line T6VS·6AL harboring *Pm21* [60] and wheat-barley group-7 Robertsonian translocation lines conferring an increased content of β-glucan [61]. Hence, the developed KASP markers specific for rye chromosome arms can be considered as co-segregation markers with valuable traits and widely used in large-scale MAS.

## Conclusions

Based on SLAF-seq and bioinformatics analyses, a total of 404 universal rye chromosome arm-specific PCR-based markers and a set of KASP markers were developed and validated. These markers can enrich the rye-specific markers and accelerate the large-scale screening of various rye germplasm resources and high-throughput detection of rye chromatin in wheat background. Two KASP markers specific for 1RS and 2RL were successfully applied in the detection of 1RS translocations in a natural population and 2RL chromosome arms that conferred resistance to powdery mildew. In addition, the present study provided an efficient strategy for wheat relatives to develop specific KASP markers, which would improve the efficiency of genetic studies and MAS for alien chromosome engineering breeding.

## Methods

### Plant materials

Five rye cultivars including Imperial, German White, Jingzhouheimai, Baili and Guyuan were sequenced by SLAF-seq to develop rye chromosome arm-specific markers. Rye cultivar Baili, wheat cultivar Holdfast, and a complete set of wheat-rye disomic addition lines of ‘Holdfast-KingII’ were kindly provided by Dr. Zongxiang Tang (Sichuan Agricultural University, Chengdu, CHN). Rye cultivar Imperial and a complete set of wheat-rye disomic addition lines of ‘Chinese Spring-Imperial’ were kindly provided by Dr. John Raupp (Kansas State University, Manhattan, U.S.). Rye cultivar Jingzhouheimai was kindly provided by Dr. Lifang Zhuang (Nanjing Agricultural University, Nanjing, CHN). A complete set of ditelosomic addition lines of ‘Holdfast-KingII’ were kindly provided by Dr. Adrian Turner (John Innes Centre, Norwich, UK). Rye cultivars KingII, CIse 1, CIse 12, CIse14, CIse17, CIse53 and CIse54 were kindly provided by Dr. Yiwen Li (Institute of Genetics and Developmental Biology, Chinese Academy of Sciences, Beijing, CHN).

The following materials maintained in our laboratory including wheat cultivars Chinese Spring, Shixin633, Shixin733, Shixin828, Gao8901, Jishi02–1, Shimai15, Kenong199, Heng5471 and Jimai22, two octoploid triticale lines (2n = 56, AABBDDRR) 09R1–38 and 09R1–100, and a wheat-rye T1RS·1BL translocation line Lovrin10, were used for the location and verification of rye chromosome arm-specific markers. The wheat-rye lines used for the verification also included wheat-rye 4R disomic addition line WR35, T4BL·4RL and T7AS·4RS translocation line WR41, 6R disomic addition line WR49, 2RL ditelosomic addition line WR56 and 2R (2D) disomic substitution line WR91, all these lines were developed from ‘Xiaoyan 6 × German White’, and previously identified by using GISH and mc-FISH analyses [20].

One hundred and sixty-one wheat cultivars/lines maintained in our laboratory and the F_2_ individuals derived from the cross of WR91 and Shixin633 susceptible to powdery mildew were used to validate the practical utilization of the rye chromosome arm-specific KASP markers. The wheat cultivar Mingxian169 without *Pm* genes that maintained in our laboratory was used as susceptible control in assessment of reaction to powdery mildew at the adult stage. All the seedlings were used for extracting total genomic DNA by the CTAB method [62]. Genomic DNA of Chinese Spring was used as a blocking DNA in GISH detection.

### SLAF sequencing, sequence comparison and rye-specific fragment acquisition

Genomic DNA samples of rye cultivars Imperial, German White, Jingzhouheimai, Baili and Guyuan were, respectively, sequenced by SLAF-seq (Biomarker, Beijing, China) as previously described with some modifications [63]. Firstly, the above five genomic DNA samples were digested with the restriction enzyme RsaI, respectively. Secondly, a single-nucleotide overhang was added to the digested fragments and the duplex tag-labeled sequencing adapters were ligated to the A-tailed DNA with T4 DNA ligase. Thirdly, the PCR amplified products were purified and electrophoresed on a 2% agarose gel to isolate the fragments with 464-494 bp in size which were subsequently excised and purified. Finally, the purified products were sequenced on the Illumina HiSeq 2500 sequencing platform (Illumina, Inc., San Diego, CA, USA).

All SLAF pair-end reads generated from SLAF-seq raw reads were filtered according to the quality, and then high-quality clean reads with over 90% identity were clustered into one SLAF locus. In order to obtain the rye-specific fragments, all the SLAFs derived from the five rye cultivars were compared with the wheat genome using BWA software [64], leaving the SLAFs with low identity less than 50%. The comparison among respective remaining SLAFs from the five rye cultivars were also carried out to finally acquire the conserved rye-specific sequences.

### Sequential GISH and non-denaturing FISH analyses

GISH analyses were performed to confirm the existence of the rye chromosomes or chromosome arms in the complete set of wheat-rye disomic and ditelosomic addition lines of ‘Holdfast-KingII’ and a set of disomic addition lines of ‘Chinese Spring-Imperial’. The root tip cells were prepared for mitotic chromosome observation and hybridization as previously described [65]. Total genomic DNA of rye cultivar KingII or Imperial was labeled with fluorescein- 12-dUTP (green) by nick translation method and used as a probe, while genomic DNA of Chinese Spring was used as blocker with the ratio of probe/blocker on 1:50. After GISH analyses, ND-FISH analyses were conducted to determine the identity of the rye and wheat chromosomes by using two combinations of oligonucleotide probes, respectively. The probe Oligo-pSc119.2–1 (green) was 5′ end-labeled with 6-carboxyfluorescein (FAM) and the probes Oligo-pAs1–1 (red) and Oligo-pTa535–2 (red) were 5’end-labeled with 6-carboxytetramethylrhodamine (TAMRA). These oligonucleotide probes were synthesized by Shanghai Invitrogen Biotechnology Co. Ltd. (Shanghai, China). The combination of probes Oligo-pSc119.2–1 with Oligo-pAs1–1 or Oligo-pTa535–2 could discriminate all rye chromosomes and 42 common wheat chromosomes. ND-FISH analyses were carried out according to the method as previously described [66]. Then, detection and visualization of rye chromatin were performed through an epifluorescence Olympus BX53 with a cooled CCD digital camera. Program CellSens Standard 1.12 (Olympus Corporation, Tokyo, Japan) was used in images analysis.

### Development and verification of rye specific PCR-based markers

According to the obtained conserved rye-specific sequences, PCR primers were designed using Primer 3 software (http://www.primer3plus.com/primer3web/) for the amplification of five rye cultivars Imperial, German White, Jingzhouheimai, Baili, Guyuan and wheat cultivar Holdfast. All the primers were synthesized by Shanghai Sangon Bio-technology Co., Ltd. (Shanghai, China). Three steps of PCR analysis were performed to develop rye-specific markers: firstly, those markers whose specific band absented in Holdfast but presented in all the six rye cultivars were considered as universal rye-specific markers. Secondly, a set of wheat-rye disomic addition lines of ‘Holdfast-KingII’ were used to assign the markers to individual rye chromosomes. Finally, a set of wheat-rye ditelosomic addition lines of ‘Holdfast-KingII’ were used to locate the rye chromosome-specific markers onto rye chromosome arms. The frequency and number of markers assigned to the individual chromosome arms were showed in a circos plot drawn by the Circos-0.69-9 software [67].

Additionally, KingII, Holdfast, Imperial, Chinese Spring, a set of wheat-rye disomic addition lines of ‘Chinese Spring-Imperial’, wheat-rye lines WR35, WR41, WR49, WR56, WR91, Lovrin10, octoploid triticale lines 09R1–38 and 09R1–100, and wheat cultivar Shixin633 were used for testing the specificity of these markers.

The PCR amplification system (10 μl total) contains 1 μl of template DNA (100 ng/μl), 4 μl 2 × Taq Master Mix (Vazyme Biological, Nanjing, China), 4 μl ddH_2_O and 1 μl primers (10 μmol). The PCR procedure was as follow: 94 °C for 5 min, followed by 36 cycles of 94 °C for 30 s, 58 °C for 30 s, 72 °C for 40 s, and a final extension at 72 °C for 10 min. The PCR products were separated in 8% non-denaturing polyacrylamide gels with 29:1 ratio of acrylamide and bis-acrylamide, and silver-stained prior to visualizing the banding patterns [68].

### Development and validation of corresponding KASP markers

The PCR-based markers specific for rye chromosome arms were analyzed to convert to KASP markers. Firstly, the sequence alignment of the original SLAF sequence with the data of *S. cereale* L. Lo7 scaffolds [36] was conducted to ensure the specificity of these SLAFs in rye genome. Then, the specific SNPs between original SLAFs or the expanded sequences and wheat were called by MUMmer 3.23 software. The 40–120 bp sequences with high consistency to wheat genome flanking SNPs were extracted and further evaluated the primer quality to design KASP markers. The KASP markers were designed using Primer 3 software (http://www.primer3plus.com/primer3web/). The wheat sequences were referred to the IWGSC Ref Seq v1.0 [35].

Subsequently, these KASP markers were validated their specificity, stability, and universality by testing 12 rye cultivars including KingII, Imperial, German White, Jingzhouheimai, Baili, Guyuan, CIse 1, CIse 12, CIse14, CIse17, CIse53 and CIse54, a complete set of wheat-rye disomic and ditelosomic addition lines of ‘Holdfast-KingII’, a set of disomic addition lines of ‘Chinese Spring-Imperial’, and eleven wheat cultivars including Holdfast, Chinese Spring, Shixin633, Shixin733, Shixin828, Gao8901, Jishi02–1, Shimai15, Kenong199, Heng5471 and Jimai22.

The KASP amplification system (8 μl total) contains 4.5 μl high rox KASP master mix, 0.13 μl Assay mix, 0.97 μl ddH_2_O and 2.4 μl of template DNA (50 ng/μl). The reaction was performed in a Bio-Rad CFX real-time PCR system (Bio-Rad Laboratories, Inc. California, USA), PCR procedure was as follow: 94 °C for 15 min, 94 °C for 20 s, followed by 10 touchdown cycles of 64 to 58 °C (decreasing 0.6 °C per cycle), 38 cycles of 94 °C for 20s, 58 °C for 60s, and fluorescence was detected using Bio-Rad CFX Manage 3.1 software.

### Assessment of reaction to powdery mildew at the adult stage

A wheat-rye 2R (2D) substitution line WR91, derived from ‘Xiaoyan 6 × German White’, was previously confirmed to confer the adult resistance to powdery mildew that was located on the chromosome arm 2RL. To further transfer the resistance into susceptible wheat cultivar, WR91 has been crossed with Shixin633 and generated F_2_ seeds. WR91, Shixin633 and their F_2_ seeds were planted in early October at the Luancheng Agro-Ecological Experimental Station, Chinese Academy of Sciences, Shijiazhuang, China. Twenty plants were grown in each 1.5-m long row, spaced 25 cm apart. Wheat cultivar Mingxian169 was planted around the F_2_ population as susceptible control and inoculum spreader. In late March of the next year, the spreader rows were artificially inoculated by the mixture of *Blumeria graminis* f. sp. *tritici* isolates prevalent in northern China. In May, when the susceptible control Mingxian169 exhibited serious disease symptoms, the evaluation of the powdery mildew reactions at heading and grain filling stages was recorded using a 0–9 scale, where 0–4 was considered as resistant and 5–9 as susceptible [69].

### Application of KASP markers in MAS

The 1RS-specific KASP marker SWK5282 was used to screen a natural population with 161 wheat cultivars/lines to determine the distribution of 1RS translocation lines therein. For the verification of the accuracy and stability of the KASP marker, the corresponding PCR-based 1RS-specific marker SW5282 was also used to identify these 161 wheat cultivars/lines. In addition, the KASP marker SWK252224 specific for 2RL was applied in detecting chromosome 2RL in F_2_ individuals of ‘WR91 × Shixin 633’. Meanwhile, the corresponding PCR-based marker SW252224 was also used to test the F_2_ population to confirm the result of SWK252224. Chinese Spring and Imperial were used as the KASP marker genotyping controls.

## Supplementary information

**Additional file 1: Table S1.** Primer sequences of the 404 molecular markers specific for 14 rye chromosome arms.

**Additional file 2: Table S2.** Detection of 1RS translocations in 161 wheat cultivars/ lines.

## Data Availability

All the data generated or analyzed during the current study were included in the manuscript and its additional files. The raw data is available from the corresponding author on reasonable request.

## Abbreviations

FISH: Fluorescence in situ hybridization
GISH: Genomic in situ hybridization
KASP: Kompetitive allele-specific PCR
MAS: Marker-assisted selection
mc-FISH: Multicolor FISH
ND-FISH: Non-denaturing FISH
NGS: Next-generation sequencing
SLAF-seq: Specific-locus amplified fragment sequencing
SNP: Single nucleotide polymorphism
