# Supplementary material for: Scale development and utilization of universal PCR-based and high-throughput KASP markers specific for chromosome arms of rye (Secale cereale L.)
Source: BMC Genomics. 2020 Mar 4;21:206. doi: 10.1186/s12864-020-6624-y (PMC7057559; doi:10.1186/s12864-020-6624-y)
Supplement: Supplementary file 1 — Additional file 1: Table S1. Primer sequences of the 404 molecular markers specific for 14 rye chromosome arms. [file 12864_2020_6624_MOESM1_ESM.pdf]

Table S1 Primer sequences of the 404 molecular markers specific for 14 rye chromosome arms

Molecular Marker	Location	Forward primer sequences (5'-3')	Reverse primer sequences (5'-3')
SW421522	1RS	GTTTCGAGCCCATGACCTTT	GTGTGGAATGTTTTGCTGCA
SW421524	1RS	TTCATGCCCCTAGTGTCCTC	CGATGGGTCACTAGGCATCA
SW421525	1RS	GGCCACGAAACCATGACAAA	CCCAAACTCCCAATATGCCAT
SW5282	1RS	TCCTATCCGCACAATCACCA	AAAGAACTCGGTGCCTCAGA
SW5283	1RS	GCACACATGACGACCTCTAC	AGTGAAGACAGGAGCCCAAA
SW5284	1RS	CCTATCCCTCTGTGGCGAAT	GTCGTGTTCCCCTACATCCA
SW5285	1RS	ATTATGACGACAAGCACCGG	TAGCCCGACATAGCCTTTCA
SW11161	1RL	AGGTTGAGACAGCGCAGATT	CAACCAGATTGGTCAAGGCT
SW11941	1RL	AATTTCCCAAATAATCCCGC	CCTGACCATATCAACCGTCC
SW12271	1RL	TATTGCCGATTGCTATGCTG	GTTGCACAACGTCATGGTCT
SW13012	1RL	TCGTAGAAGTGTTCCATGACAA	TGCTTGTGTATGGGCTTGAA
SW15320	1RL	GCGTGCGTAGAGTTGTATCG	GTGGAGTTCGTGAGGCAGAT
SW17300	1RL	CCAATGCTAGTTTCCTTGGC	TGCATGCAAACTGTGAACAA
SW19081	1RL	GGAGGTGCGGATATCAGATG	TTCGGTGATCACGACTTATGA
SW20160	1RL	GCCCAAGTAGTCTCTAACCGA	TCGTAGTAGCGCCTGGGTAT
SW21253	1RL	CACAAGTAGCGGACCTCCAT	GAGGCATTCCTCTCCATCAA
SW23822	1RL	GTTATGGGAATTTATGGCCG	AATACGCCGAATGGATAGGA
SW25074	1RL	TGAGGAGGAAAGGAGCAAGA	TCTGGGTTGTGTTTGTTCCA
SW25581	1RL	GGCAGAACAGTGCAGTTGAA	CGAGTCCGGAGATTGTTGTT
SW30220	1RL	GGTATTCGTTTACCACCGGA	GCGTAGCCGTTACTATGTTCG
SW34440	1RL	GGAGGCACACATAGGCAGTT	TGCGTATGAACCAGCATCTC
SW41722	1RL	TGCTGGTTAGGCTGGGTATC	GTTTCGTTCCGTTTGGACTC
SW44747	1RL	TACACTCAACGAGCCCAGTG	CGAGACAAGAAGAAGGAGGG
SW48988	1RL	CGGCGGATAGGTATCATCTC	TCACACCACAAGAATGCAGA
SW51098	1RL	GGTGCAGACATAACCGGAAC	GGTTGTTAGGCCAACTCCAA
SW54980	1RL	TCAACCATCAACTTTGTGCC	TTAGAAGAGGAACCGCCAGA
SW57819	1RL	GCCCAAGAAGCGTCTATTGT	CCATCCATCACATCATTCCA
SW66846	1RL	TGTGGATCGGAGTTCATGTG	CACGAGGCTCAAGCAATACA
SW34558	1RL	ATTCCCATGCATTTACGAGG	TGCTAAATGGAGTCGTGTGA
SW35806	1RL	TGGCTAGGATCAATCACCTTG	ATGGATGGATTTCAGATCGC
SW42759	1RL	ACAAGGACACCAAGGTGAGG	GCAACACCGGTAGTAACATGAA
SW54980	1RL	TCAACCATCAACTTTGTGCC	TTAGAAGAGGAACCGCCAGA
SW61869	1RL	TGTAGCCCTCATTTCGAAGG	TATTCCCTCCTCCGTGTGTT
SW62979	1RL	TCGAGTTGATGAATCGGCTA	AATCACAATCGCAGCAACAC
SW65093	1RL	CTCGCTGGGTAGTTGTTCATC	TCGGGAGGTTACAACATGAAA
SW66497	1RL	CTCGCATGTATCTCTCGCCT	ACAAGCGTAAACTGCCATCC
SW8593	1RL	CCTGCAAGTATCCATCCACA	GGCGACATAGGTCTTCATGG
SW34440	1RL	GGAGGCACACATAGGCAGTT	TGCGTATGAACCAGCATCTC
SW34726	1RL	TAGTCCATGACACCGTCAGC	GGAATATGGCGAAAGCACAT
SW35363	1RL	GGAGTTGAAATCCGTCTTGC	CGTGAATTCGGTGATCAGG
SW35605	1RL	CATCAGTGTCAACCACGACC	CACGCATGCTTGCAGTAGTT

SW621	2RS	GTGACGCCGAAGCAGAAC	CCAAACCGACGCAAGAAC
SW625	2RS	TCCTCGTCTCATCCAGCATC	TGCACAACCACAACCAAACA
SW9021	2RS	GCCCCATCTGAGCAAATACG	CCCTATGGTTGTGGTGGTCT
SW9022	2RS	TAATGCCCACCTAGACCCAC	TGTCAGATGCACACTCACCT
SW10343	2RL	TTTACAGTCCACCAGCCACA	AGTGCGAATTTGAATGCTTG
SW10636	2RL	ACCCTTGGCCTGACATAGTG	TGCAACCACCTGAAGTAACG
SW11163	2RL	AGTTGCGGAATAAACGGAGA	AACGCGAATCAGACAATCG
SW12881	2RL	TCACACGGGTATCTCTGCTG	TGTCTGATGCACCGATCAAT
SW12924	2RL	GACACCAAAGGGAAACCTGA	AACTTGCCGTGCTTACCTGT
SW14174	2RL	TCTCTTCCTGCGAGCATCTT	ATAGGAGGAAGGAGGGCACC
SW14345	2RL	AGCGCTACAGTTTCCGTTTG	ATGGCGGATGCCTATATGTG
SW14686	2RL	TGGAGTCCGTTTGATATCCC	CAGACTGAACGCGCCTAAG
SW14843	2RL	GTCCGGCTCTAACAACATGG	AAGAGATCCAGGCGAGACAA
SW14928	2RL	ACCACTCCTGAGCCAAGCTA	CGACCCACTTCGTCTGAACT
SW17412	2RL	GATGGCCACGATAGAGGAGA	TAAACACTTGCGCTTACCGA
SW17933	2RL	ATCAGCATCCACACTCACCA	TGCAGTCGCATTAGCTTCAG
SW18917	2RL	AAACCAGAGGGCTTAGTCCA	CCTTCGGGTAACTTAGGCAA
SW19466	2RL	GCCATCAGTGGAGAAGATCAG	GCGTGATGGAACATGACAAC
SW20081	2RL	TAGGCAGGAGCAAAGAGGAG	GGCAGGTTACAGTTGGACTTG
SW201658	2RL	GCAGTGTCATGGCTTGGTC	TTCATGTGCGAACACTAGCC
SW20934	2RL	ACTGGCGGATGATACGGTT	AAGGCGATTATGCAAGTTGG
SW23298	2RL	AATTTGGCGCTACGAAAGAA	TGTGTTGATAAACGCTTCCG
SW22374	2RL	AATATCTGTCGCAAGGGACG	TTTGCATTGTTTGGACGTGT
SW24019	2RL	TGGTAGGTTTGGCTACGAGG	CGAGAGACTGAGCCGACATA
SW252224	2RL	TTCAACACCAAGAGAAGGGAA	CAAGCAACGAAATTCTAAAGCA
SW25550	2RL	AAATGAGATTTAGGCACTGCAA	CGCACAACCTTGTTTCCTTT
SW277397	2RL	GTGGTTGCTACCTGCTACGG	ATAGTGACGCTTGATGGCG
SW28671	2RL	ATAGTCCGGCGTGGTTTACA	GGTCTCTGTGCTGTCCACAA
SW29856	2RL	CCTCTGCTCTAACCCTGCAC	GGAAAGAGCCTACATGCTGC
SW30156	2RL	AACTGATCGCTGGATTGCAT	CCATTACGACAGGTAGCCCA
SW30176	2RL	TTGAAATCCCTTCTGCTTTG	AAACGGGATGACTTGGCATA
SW31546	2RL	GCCAATTAGGAGGTGGTGAA	AGAGGTTGGTTTCAGAGGCG
SW31728	2RL	GTCTATTTAGCGTCCAGCCC	TCTGGATTGCGTTTGTTTCA
SW37832	2RL	CCTGGTGACATATTGAACACAGA	ACCCGTAAGTTTGCAACCAA
SW18234	2RL	TCATCCCTTCTCATGAAGCAC	CTCCGACCGAGCTCTGTTAT
SW20934	2RL	ACTGGCGGATGATACGGTT	AAGGCGATTATGCAAGTTGG
SW22600	2RL	GAGAGAATCCGCGACACAGT	GGGGGTGGTAACGTCAAGTA
SW54247	2RL	GCTATTTGTTGGCCTTGACC	TTTGCGACAACTCCTTCAGA
SW131261	2RL	TTGTGGCATTCAATCAGGAA	TACGAACCTGCAGTGGATTG
SW13924	2RL	CAATATCTCCGACAGGACACG	CAGGGTTCAAGCGGTCATAC
SW29004	2RL	CAAATCTCATGTGCCACCAG	TGCGAGTCAATTTTTCTCTCC
SW88434	2RL	AAATGAGAAGAAAGACACGGG	CAAAAACTGATTCAGACCCACA
SW43820	2RL	CGTCTACGCTTAACGCAACA	CCTTTCCCTACACCTGAAACC
SW53444	2RL	TAGATAGCCGTTTCGTTGGC	GCCCGAACATTGAAACAACT

SW55241	2RL	GGCATCATCCCATCAACAGT	ATCTATTGTGGCGCTGCTTT
SW62074	2RL	GGGCCATACATGGGATAGAA	TGTTCCACACGTTGTTGGTT
SW64357	2RL	CTTCAGTCGGTGTCCTGGAT	AAGATATCAACCGACGGACG
SW66023	2RL	CTCCGCCTGAGATGAAGTTT	GCGATTAGCTAGCAGCTTGG
SW738122	2RL	GCACAGAATCGACAGAACCA	CGAATTATGTATCGCGGTGA
SW74759	2RL	ACCATGCAAAGTTTCATCCC	GTGGAAGTTGCTCGATGACA
SW82535	2RL	AGCAGGAAACGAGGCAATTA	GAGCTAGACAGCATGGTCCC
SW828024	2RL	TCACCGGCTATGTGATGCTA	CCGAGTAAATTTCATCAGGAGC
SW91426	2RL	AATGCTTTCCAGTTTGCAGG	GCCTGTTCATCAAGTGCAAG
SW9666	2RL	GCGCACTCTATCACGCTACA	ATGAAGCAGCGAGCAAATCT
SW33661	2RL	GACAAGGGAGAGTTTCGTCG	TAGGAGGAAGGAGGACACCG
SW33999	2RL	CCTTCCCTCGTTCATCCTTT	TTCCCACCACTAGGCTATGC
SW34109	2RL	ACTTTGCTGCATCACCCTTT	CCTCTTCTCTTCGAGCATGG
SW11736	3RS	GTGGCCCGAACCAATATAAA	ACGCATGCTATTCTTGCCTT
SW14612	3RS	CGTATTCTCCTCATGCCCTC	CTAGTTCCCAGGACACCGAC
SW17838	3RS	AGCACCGCCAGTAGTTCTTC	GGTCTGGCAAACACACATGA
SW18339	3RS	TGCATGCAGATGAGATCAGA	GATGCCATATTTACCCACGC
SW18416	3RS	GGACAGTTTGCTGTCTGCC	CTTGGCTCTCGAAACCTCAC
SW188318	3RS	ATCGGATTGAAGGTCAATGG	TAACAATCAGTCACCGAGCC
SW18833	3RS	TGGACTCCGTTCGATATTCC	CCTGTAAAGGGACTGCAAGG
SW20265	3RS	TGAGCTTGTTGCTCTTGTCG	CCAATCCAGGGATGACACTT
SW232058	3RS	AACCACAGATTGGTTCACCC	ACGGCATGACTTAAATCGCT
SW28002	3RS	GGACTGTTGCGTGTTGATGT	GTGCCGTTACCAGATCGAAT
SW31692	3RS	AATGTTTGAGGTCTGGTCGC	TGTGCAACAAACTCTCTTTGG
SW48367	3RS	TTCCAACATCGTATGGGAGTC	ACCTTCTCGTGTGATTTGGC
SW52416	3RS	GGGCTTGTGAAATGTCTTGAA	GCACAAGCATTTAATGTGCAG
SW25383	3RS	GGATCCTTTCAATCCACACAA	CTTCACATGTGGGTTGCATT
SW16446	3RS	TGTCATCCAGCCACAAAGAG	GTGGCCCGAACCTAGATAAA
SW54575	3RS	TAGCCCATTGTTACCAGCAA	ACCCATCAGGAGATCAGTGC
SW33773	3RS	TCATAGTGGACCACGACCG	TGCCCAGAAATTTCCTTTGT
SW43890	3RS	TTGCTCATCAAGTGCTTCGT	GTGACATGGGTGTTTATGTAGC
SW79826	3RS	GACCAGAGATCGCCACAAGT	GCTGGGACCATCTAGCAATC
SW82984	3RS	TTATCCCATTGCAAACCATGT	TGTAAGGTTCGTCTCCTGCC
SW33512	3RS	AACAACATGGAACAATAAATCTCAA	TTGATTGGCAAACCAAGAAAG
SW33555	3RS	CATGAGGAACCAAAGAAGGC	TGGGTTTGTTGCACCAATTA
SW34287	3RS	TCCGTGGTTCTCAAGTCTCC	ACATCAACACCATTGCCCTT
SW11509	3RL	TCGATCTAGCCCATGTGTGA	CTGGTCAGGACTTTCATGGC
SW12117	3RL	AGCATCTGAGGCCAAGAGAA	CCACCTGAAAGCCATAAGGA
SW12812	3RL	ATGCCGAGCTTCGATGAAT	CTTCCTTTGTTTCAAGGCCC
SW15063	3RL	TCCTTCTCCCTGATTGGTTG	CACCAAGTGTCGTTGTTTCG
SW19349	3RL	GTGGGAGGCTTGTCCAAGTA	ATGGCGAACAGATCCAAGAG
SW21484	3RL	CTCACCAAACCAGTCAACCC	TGCTGTTTGGATACGGTGTG
SW25919	3RL	TGGCTAAATTGCTTGTGTGC	CCCTAGTCCATGACACCGTC
SW27284	3RL	TGTTGACCTACCCTAGGTAATGC	AAAGGTTCCACGTCTGCATC

SW29102	3RL	AAGGAGTGAAGTCACCTCGG	GCTGAAGATGCTACAGGCAC
SW30967	3RL	TGGAGCATCCAAACAAACAA	AGTCGGCGTCGTATCGTATC
SW31167	3RL	AAATTTCCTCCTGCCTGAGC	TGAGGAGACGTGCTCTGTGT
SW32082	3RL	CTTGACGCATGGAACAAGTC	TTCCTTCTCGCTCTCCAGAC
SW37185	3RL	TATGCATGTGTGGACCACCT	ATACTAGCAAGATGCCCGTG
SW39072	3RL	GAACCGATGAATCAGCCAAT	ACCAATGAAAGTGAGGCACC
SW50241	3RL	ACGAGGTCACCCTAACTCCA	AACATTTGTCGAACAAGCACA
SW69637	3RL	GAAGTAGAGGTGCATCGCGT	GGGTTGTTGCCACCTCAG
SW94464	3RL	GGATGGGTCTTGTCCATCAC	GCGGCATGCTATGGCTATT
SW17835	3RL	CACAAATTTGCAGCCGATTA	CTGCTTGCAACTTACGGGAT
SW10727	3RL	CGGTCATCGCGTAGGAGT	CAGGTATTTAGAGCAGCGCC
SW22986	3RL	TGGACCATCTTTGTTGCCTT	CGTTCCTGTGTCTGTCCCTT
SW44078	3RL	AGGAGGTGACGGTAATGTGG	GATCAACTCACAAATGGCGA
SW45736	3RL	GTCGGCAGAAAGAACGCTAC	ACAGTGGGAACCTCTTGTGG
SW538142	3RL	TCTAGTCGTCGTGAACGCAT	GCTTCATCTTTCCTTCGGTG
SW76494	3RL	GCATGGTAGCAATGCCATAA	TTGCTTCAGCATCCAGTTTG
SW845002	3RL	CGATTGTTGGCAACTTTCCT	TTCTTGCATCTGTGACCCAA
SW10817	4RS	CACACAAAGCACTCCTACGG	CCCTCCACAACAGCCTAACA
SW11920	4RS	ATGATGAGGAACGTAGCCGT	CCAATCAGCAGCTTGATCCT
SW12136	4RS	TTGTGCAAGAGCTGTTGGTT	ATTGTGGTGTCATGCAAAGC
SW12286	4RS	CTTACTATGCCTTACGCCCG	GGTGCTAAGCCTTCCACGTA
SW12489	4RS	TAGAAGACGGCGGTGAAGTT	GATCCGCCTGTGGTTTATTT
SW12979	4RS	CGGTTGCAAAGGATCAATTC	GTATCCGGGAGTTGCACAAT
SW14934	4RS	GATCTGTGCCTGTGTGCAGT	TGTAGAAGGGTTCGTCTCCC
SW16598	4RS	AGGAAAGGAAACGAAAGGGA	TATCACAAGCGCAGTAAGCC
SW16760	4RS	GCTTCAAGCTGATGCAGATG	TCTCATGGTCCAAGGAAAGG
SW17280	4RS	TAAACGTCAAGCGTGCGTAG	CAAGGATAATTCTTCGCGGT
SW18800	4RS	CATGCAATGTCTGAGGGTTG	CAGACAAAGGCGTCACAGAA
SW24500	4RS	TTGCCTTTACATTCCTGTTGC	GGGTGGCGTGAGTTGTAAA
SW24984	4RS	CCACACGACATGCGAATTAC	TTGTTGAGCAACCTCAGCTC
SW25192	4RS	TGATGAGTTGGATGGAGCTTT	CACTGTATTCACCACCTCGC
SW30487	4RS	GCAGGCAAGTAACCAAGGAA	AGGGCACATAACTTGGGAAA
SW31722	4RS	GCCGCTTTGTTGAATCTTGT	AGATGTGCAAGAGCTTCGGT
SW27567	4RS	CTGTAGGTTCTCCCGTGCAT	TGCCCAGAGTATTCGAAACAA
SW84885	4RS	CGACCAAGAAACCCAAGAAG	CTCTCCCTCCTCGTCATCTG
SW93843	4RS	TGGAATCCTATGTTCATGCCT	CTTCCTTTCCGTGTCCTTTG
SW11734	4RS	CGCCTAGAATGATGCTTTGG	AAACAGCGGGATAGAAGCAA
SW38543	4RS	ATGACCTCAGAGGCTTGTGC	TCAAATTGGTATCGTGACATAACTT
SW4597	4RS	GGGAATAGGAGCCCAATCAT	CCTGCCAATGCACACTTTAT
SW71465	4RS	GCCATATTCCAATTTGCGT	TGAAAGACAACTGCGTGCTT
SW750420	4RS	GGATGAAGTTGGAAGTTGGC	TTTCCAGATGCTGTCGGTTT
SW105488	4RL	CATCCAAATATGAATGTGCCTC	CAGCTGCCACGTCTAGTGTC
SW10788	4RL	ACAAGCTTGGAGCCAAAGAA	GGAGATGGGCAAGAATTTCA
SW12205	4RL	TGGCCCGAACCAATATAAAC	GTGTATGGCAAAGCCCATTT

SW12831	4RL	TCAAATGCACTTGGCAATTC	TAAGAGCGGGATACCAGACC
SW13716	4RL	GTTAAGCATGCGGACCCTAC	TTGCTTGCTTGTTTGGTGTC
SW15200	4RL	TAAACGTCAAGCGTGCGTAG	AACACATCCTCGTCTCCACC
SW15882	4RL	TCCGTTGGAAAGCTATGGAC	CCCGTCAATGAAAGAACGAT
SW16368	4RL	TGATCTCGTCTGGTGATCCA	CTGCTTCCTGCTTTGTTCCT
SW16471	4RL	AGGTAGACCCAACTCGACCC	TATGGTCGCGTTTCTGAGTG
SW16729	4RL	CACTCAACACGTGATTTCGC	CGCAGAATGCGAGACAATAG
SW16910	4RL	ATTTGCCTTCACCCTTCCTT	CTTCCGGTGTCAGTGATTTG
SW17800	4RL	CTCGAGAGCGCTTTCCTTTA	GTTCTCTTGAATGCTTCCGC
SW18624	4RL	GCTGGCCATGCACAATAGTA	AGGAGGAGGTGGGATTATGG
SW19079	4RL	GTGCATCTCAGCGAAGTGAG	ACGACACAGAAGCCAACACA
SW20928	4RL	ACATGAGTCACGAGGGCAAT	ATGGAGGCTGATAGTGACGG
SW23743	4RL	TAGTCCTTGACGAACGAGGC	TTGTGCACGCTGCTCTACTT
SW25323	4RL	GTCAATCACTTAAGCGCCGT	TGTGTGCCAGCTATTGATCC
SW26053	4RL	GTTTCCACGCGGTGATAGTT	GCATTTAGATCGCTGGAATGA
SW26269	4RL	GCCAATAATGTCCTCGTGCT	ATGCTCCTGCCTCCTGTAGA
SW26399	4RL	GCAGCAACATCGTCTCATCA	TCATCTCACTCGATTCGCAA
SW30649	4RL	TCTCTGAATGTCCGAGACGTAA	CACTGTAGCCACGCCCTTAT
SW31024	4RL	ATTGCCTCTAGGGCATATTTC	TCATTAATGGAAAGCAATGGG
SW32938	4RL	CCAATAACGCATGTGGTTGA	TTGCGTGGCAAATACACAAT
SW33259	4RL	CCCAGGTGTGCTTCTTTCAT	ATTCATCAACCTTCGCTCGT
SW34086	4RL	ACATACGGAGCAAATGAGGG	CCAGTTCCGGCAAACTCTTA
SW36796	4RL	TCTCCTGCTCAGACATTAGCC	ATAGGAAGAAGGAGGGCACC
SW49509	4RL	GGGACGTTGATTTAAGTGGG	GCCGTTAGCAATTGACACCT
SW51004	4RL	TCCGTCCGGAAATACTTGTC	GATTCTCCATCAGGATTGGC
SW58789	4RL	AAGCACAAGGCTACCAGAGC	TGAGATGATGGGTTGTTGGA
SW8570	4RL	ATGAGCCGATTCATCTTTGG	TGATATCGCATGTGAAGGTTG
SW10099	4RL	CACAACTTTGATGTGGCACC	GGTCGGGAAAGTCGGTTATT
SW24301	4RL	TCAACTTCCAAGCTTCAGCA	ATCTGGTGCATGAGTTGGCT
SW25001	4RL	AAGGTGTGATGTGCAAATGAAT	CAAGTTGGATCAACATGCTACTG
SW21934	4RL	AGTTCCTGCACCAACAAACC	ATCAATGCAAGCAATGGTGG
SW23640	4RL	ATACTCTTTGGCGACATGGG	CTGCTATCGCCTCTTCAACC
SW34707	4RL	CTCTGCCGCTGGAGTATGAT	ATGCAGCATGCTTCCTCACT
SW47281	4RL	TTTAGGTATCGATGTGCCCTG	TTGTTGTGAGTGGGAAACCA
SW57799	4RL	TGAGCAGTTTGTCTGCCATC	TCGACACCTTATCCAATGTTTG
SW61253	4RL	GATGGGCTCGTGAAGGTCTA	TCTGCACGAGCAAGTAGCAC
SW61663	4RL	CGGGACTTCCCAGAGGTTAT	AACGGAGTTTGCTCACCACT
SW63704	4RL	TGCATGGAAACTGGAAGAGA	AGGCCAGTCTCACTTCTTCG
SW67058	4RL	GATGAAGTTGTCCGAAACGG	AGGCGGGCTATAAGCTCACT
SW7531	4RL	TTTAGGATGACAGTGCGGTG	TCAAAGTTACACAGCCCACG
SW7728	4RL	TTTATTAAAGGACCGAGACACTAAA	CCGAGTTTCACCAACTGGAT
SW82649	4RL	TTCTTCACCACCTCCCAATC	CGACTCGTTGGTGGTAGCTT
SW84378	4RL	GCATGTATTGGGTTCCATGA	TGAGGAGGTGCCATGTATGT
SW342000	4RL	TGGTGCAGATAAGAGGGAGG	GATACCTCAGGAGGCCCTGT

SW34247	4RL	TTTAACCATGAAGGGATTCACC	GTAGACATGCGTGGTGGCTT
SW35298	4RL	GTAATGTTGCCCTGGAGAGG	CAACATCGCACGACAAGTCT
SW29842	4RL	AACACCGCTGGTATGGAAAC	ATAGGCATCAAACAGCTGCC
SW11196	5RS	GAAGCTATTGCTGGTCTGCC	GTGGCGAGTGTAAGCCAACT
SW13286	5RS	TCGACCATTTCGAGACTCCT	CAATGCAGGAGATGGTTCAG
SW14110	5RS	ATGGACGAGGCACAACTTTC	TGCCTAGTAAAGTGAGGCCG
SW14274	5RS	GAGCTCGGTGAGGATAGTGG	ATGGAGAGAGTGGCCGTATG
SW14706	5RS	CACTGATGCACAGAGATCGG	AGCTCAACCAATGAGCATGT
SW17305	5RS	TGGAGACGGATTTGGATCTT	ATGCCACATCACACTCAGGA
SW17749	5RS	CGGTCACCCATCCTGAAAT	GCCCTCCGAGAGGCTAATAC
SW190654	5RS	TGAATCAGCGTGTGTTTGGT	TTGCTGTGGAAGAATACTGGAA
SW19518	5RS	AATTTGTAACCCTGGCCCTT	ACCGCTAGGAGTAGGGTGG
SW19768	5RS	GCCATGGTGAGTCATTGCTA	ATCCTCAGCCTACAAGGCAA
SW21003	5RS	TACTAGCCGCACGTATGCAC	GTTGGTTGTGCGGAGAAGAC
SW21839	5RS	CCAACACTCATGAGGAGCAC	ACCACCTATCAGAACCACGC
SW25386	5RS	CTCTCAGGTGGATCTTGGCT	GGACCATTCGAGACAACCAT
SW28135	5RS	GCAAACACCTCAGAAGAGCA	TCTGGTGACGATGAAGCTGA
SW32825	5RS	CAGAGAACACCAGCGATGAC	CCTTAAACCGCACCTAGCAG
SW32972	5RS	ATGGTGGCTTGGCCTTATAG	GCCTTCCTACTCCCTCCATC
SW33277	5RS	ACTAGTCATGTAACCCGCCC	TGCATGGTTGGATGCATATC
SW34293	5RS	CCTAAGGCCTGGGAGGTAGT	AAGGCGATAGAAACAAGCAAA
SW41981	5RS	GGCTAGGTGAGTGATACGCC	GAAGAAGAAGGGCACCGAG
SW48528	5RS	GCAATTAATGCTTTCACATGG	CGATACTCTTTGTCTGGTAAGCAA
SW21921	5RS	CCAGGCATGAAAGAACAGGT	CCGATCGACCTCTTGATGTT
SW35699	5RS	TACGAGTTCAAGACCCTCGC	GGGCCAGGATAGCATTCATA
SW37390	5RS	TGAATCCGCAACAAATCTCA	ACATGCATGCCAGATAAGCA
SW37506	5RS	CGAGGGTGCTATCATGGAGT	CACACATGGTCAAGGAATGG
SW42474	5RS	TCGCTCAGAACTACCCATCC	AACACATCGTGAGTTGTGGG
SW42936	5RS	TGCAAACTAAAGAAACGTTCACA	CATGCCCAATTCAACCTTCT
SW46724	5RS	GATCTACAACGCCATGTCCC	TACAGCTTGCACAAGGATGC
SW6746	5RS	TAAGTCGTCCAGTCTTCGGC	GGGAAGCTTTCACAACCCTT
SW75152	5RS	CAAGTGGTCCTATGGCAGTG	ATTCGCGTGATCAACCTCTC
SW9823	5RS	CTAAGCATGCCTGCGACC	TGATGTCGTTGACAGCCTTC
SW10936	5RL	TAATCCACCACACAGCTGGA	AGCGTGATCTTGGTGAGACC
SW13514	5RL	TCAAAGGCGAGCATACGATA	AATAAACCATTCGCATTGGG
SW17032	5RL	GAGTAATTGCGAATTTGAGGAA	CAACATCGCACGACAAGTCT
SW17531	5RL	CTTTCAGCTCGGCGTCCT	TTTGTCTCTGAGTCGCCCTT
SW18242	5RL	GATTGGAGACGGGAACAATG	TACCGCTTCTTCTCCGCTAA
SW20508	5RL	TCGGATACTTGATCCCTTCG	CCAGGCCAATCACATAAAGG
SW21346	5RL	CTAGGCCAACCAAAGTGCAT	TCTTTGGAGACCTCAATGCC
SW21725	5RL	CGTTCGTGCTTGTAGACACA	AGCGATAGATCCTCACGTTTG
SW22058	5RL	GGAGTCAAGGTTGCTTTGGA	TGTCTGCTTTGATGAGGTGC
SW23984	5RL	AAATCTCCTCGTTTGAAAGGAAT	TAGAGCCCGAGCAAACTTGT
SW24289	5RL	TTGGAAGAGATGTGTGCTGC	TCCCTCCATTCTTAAATATTTGTC

SW28387	5RL	AGGCGAATATTGAGCCTTCA	CGCGAATCATATGAACGTGA
SW29815	5RL	CACACTCTCACCCTTCCACC	GGGATTTGCGTGTAGTCTCC
SW29849	5RL	CGCTGCCTCTACATCACAAT	CATGCATACTCTTTGTTCACACAC
SW30661	5RL	TCATACCTCGGTCGCATACA	CGTCTTCCAGCAGGTGAAAT
SW32224	5RL	CTTCTGCCAAAGATGGCACT	TTAAGCAGAAGTGGTTGGGC
SW27082	5RL	ACTAACCGCAGAACAGCCAT	AACGAAAGAAACCTTGCAGC
SW37355	5RL	TTGGTCATCTGTCTTGAATTGC	TTTGCTAACCCTTGGTGTTT
SW40625	5RL	CCTATGCACCAAATGCAATG	GACACACTCTCCCGCATGTA
SW49398	5RL	AGGAGATTACGGTGGCCCTA	AAGAGCTCGACGAGACGAAG
SW53780	5RL	ACTGAAGACCATCACCGACC	TGACTCAAAGATGAAGACGGG
SW67956	5RL	CACTCTTCCCGCTCATTGAT	AGGAAGAAGGACTGCACGAA
SW83389	5RL	CACTCGCGAACAACCTAACC	TTCATCCGATCAAACCTTCC
SW20375	5RL	CCGCTGGAGCCTAATTATCA	GATGCATACCGTATTTGCCA
SW14478	5RL	ATTTAATGGCGTGGAGATCG	ATACAGCTGCTCAAGCCCAT
SW37703	5RL	ATGTTCGGTTTCCACTGACC	TAGCTGCTCGACTTATCGCA
SW566735	5RL	GCGACTGAGTGTTGGTTCAA	TTGCGTATTTGCGTTCCATA
SW59445	5RL	CCAACGTGTCCAGGTTCTTT	CTCCAAAGCTCGTCCTCG
SW73318	5RL	GCATTTGATCCAACTCAGCA	ATGGAAGATGGAGCCACAAC
SW76557	5RL	TGGCACTAGCGTTCTTCTCA	AGGGCAACGGTGATCTTTC
SW33888	5RL	CTTGTTGGGCATTCTTCAGC	TAGGAAGAAGGAGGGCAAGG
SW35130	5RL	CTCCTCCACCTCAGCAAAGA	GAGAGAAACACGCAACCACA
SW35167	5RL	TTCCATAGGGCATGTTGGTT	CAGCCATCATTATTCAGGCA
SW89201	6RS	TCAGGTCCTCTGCATTCTCC	CTCGTTGCTCTGCTTCTCCT
SW25368	6RS	CTCTCCCTCGCTAACTGCC	TGTGTGTGAGTTGGATGGGT
SW22057	6RS	GAAGAGGACCGATGCCACTA	TCACACTCCGGACAATGCTA
SW231201	6RS	GGTTGTTGGAAGATCACAGGA	AATTGCTCCTCGAGCTGTGT
SW12084	6RS	AATAAACCATTGGGTGCATGA	TGCAGTGCAAACCTCAATTC
SW12175	6RS	AGATGTCCCAGTTCTCACGG	TAAGAGACCGTCTTGGCCTG
SW17942	6RS	GGCCGGAAACTGTAGGAAAT	CCTCCGGTGTCAGTGAGTTT
SW18911	6RS	TGCCTGCTTCTATGTTCACG	GGCGGAATGAGAAATGTGTT
SW35807	6RS	AAGCCGACTTTCAATCAGGA	TCCGGATGATCAGTAGTGGA
SW4837	6RS	GCCGGGATAGGAGGAGTAGA	GTGCCACTGTGCAACAACTT
SW51347	6RS	TTTCTTTGGCAGTTGGGTTC	GTTGAAAGAAATGGCTCCGA
SW7073	6RS	GCACGTGCACACACATACAC	GGGAGGACGTCCCAAGAATA
SW88158	6RS	TCCACATTCACAGGTTCTCG	TGGTGTGAAACCGATATTGC
SW92529	6RS	ATTCCTGCGTCACATTTCTG	GAGCTACGGCTTCGGACAT
SW35264	6RS	ATCGCTGCAGCACATTGATA	TGTGTGCATTTGAGACCTCC
SW38534	6RS	ATCTCAACCATGCCCTTAA	TATGTGGCTCATCCTTGC
SW19719	6RL	CCAGCATCTGCAATACATGC	TTAAGCGCGACGAGGTTATT
SW21690	6RL	AATGCCGAGCCTAATCTGC	TTTACCTGCAACCCTTCACC
SW25770	6RL	TCGAAGTATGAGGGTTTCGG	TACTAATCTGGCGCCGTAGC
SW26615	6RL	GCCAAGGTTGTCACCTCAAT	TGAACTGGCCATTTGCACT
SW194725	6RL	TCTCGTTCATTCATGACCAACT	CTGCAATGAACACTGGCAAT
SW4106	6RL	CCAGGACATTCCTCCATCAA	TGCTAGTTCCCTCTGATCCC

SW41908	6RL	CTTGCCTCGTTTGGCTTTAC	GACACCACCTAGCGACGACT
SW45156	6RL	TGAACAAAGTGTGCAGAGGG	CCGCTCGTTGCTATTCTTCT
SW55879	6RL	AAACCCGAAAGCACCCTAAC	TAGGAGGAAGGAGGACACCG
SW66094	6RL	GGTCTCAAGCCAATCACAGG	ACGAACTGAACACAACAGCG
SW7244	6RL	ACGGTATCCAGGAGTTGCAC	TGGCAATACGTGGTTCTTGA
SW89869	6RL	ATGGGACGGAGGGAGTATCT	TTTGAAATGCGCAAACATTC
SW22063	6RL	TCTGCTTGATGATGATCTGCTT	TCCGCAAACCCTAACATTTC
SW30533	6RL	GCTCCATGACGACCTCTGTT	GGAGGATACCCTTGGTCCTC
SW22810	6RL	GAATGGGACCAGAAGAGGGT	CGAGGCTAATCCACCACACT
SW25803	6RL	GGCCATGTCCAGATCAGAGT	GCTTCGAGATTAGATCGGCA
SW22465	6RL	CATTAGCAAACAAGCAGGCA	CTAAGTGTGCGTTTCCTCCC
SW30582	6RL	AAAGCTTGAGAATGAGATGTTGA	TTTGTCGAGAGCAACAATGG
SW31418	6RL	ATTAGAAAGGCCATCCTCCG	TGTTTGAGGTTATGCCAGGA
SW10933	6RL	GAGCCCTACGTGTAACCTGC	TTATTTCTGGACTCCGTGGC
SW12143	6RL	TCTTTGTTCCATCGCATCAG	TGGAATTGTAGCAGTGGAAGC
SW13567	6RL	CCTGGATTGATTGCTTGTCA	ACAGTTGACTTGAGCGGCTT
SW16105	6RL	GCTAGCTGTGATGTTGACGC	ATCGATTGCATTCCGTCTTG
SW18098	6RL	TGCCACTTGTCTTGCTCATT	GGGTCAACATCATTTGGGAG
SW18485	6RL	GTGCCGGTTAGATCGTGG	AGTAAGGCACATCCAGGCAC
SW20073	6RL	TAGACGCCTAGGTTTCCGC	ACGCCCGAACAACAAAGTAG
SW2023	6RL	CCCTGGTTAGGAGGACATCA	CTCCTGTTGATTCATTGCCA
SW39134	6RL	CGGCAATAACTATGCCGATT	AAGACACAAGCCAACACGAA
SW49150	6RL	ACAAAGCAGGGCAGACAAAT	GGGAACAATGTCACCGGATA
SW5009	6RL	AGCTCTAACCAAAGCCACCA	ACCTTGTTGTGGTGGAGGAG
SW53715	6RL	CTGTGGCTGGAGATAGGAGC	AGTGACCCATCCATCAAAGG
SW54466	6RL	TTGAACCCATATTTGTTTCTCTACC	TTGATGCAGATGTGCAATGTAG
SW5483	6RL	TGTTCCATCCCAAGTGGAGT	ACATTTCTTCAAATGTCCCACT
SW5925	6RL	ACCTCACGTTCCTCTCATGG	CCACCTCCAACACAACTCCT
SW62508	6RL	AATCCGTGTCCACGCATATC	CCAACCAATAGCGTTTACGTG
SW62883	6RL	CGTACGTCATACACGCAGGT	GCCTCATGAGAGACGAGACC
SW66607	6RL	CCTCCGTAAAGAAATATAAGAGCG	TCCCAACAGAAGGAAACACC
SW84137	6RL	AATACAACGATCGCCCTCTG	GGATCGAGCATGACCTTGATA
SW34052	6RL	ACCACTTCCTGCTCTTCTGC	TTGTTGCTTCCACTGACTCG
SW34443	6RL	CGTGACGTTCTAACTCGCAA	GCAGCATGCATGAGTAAAGG
SW35205	6RL	TGCTATGCATGTTCCTTTGC	TGAAGGGAGGCTATCCAAGA
SW27156	6RL	ATTGCAAAGACACCTTTCCG	TGTTCAAGATACTTCCAGCCG
SW13519	7RS	CTTTGTTGCATCAGAAGGCA	TTCACACTTGAGTGGTTGGC
SW148481	7RS	AAACACGCCTAAACCATCTTATC	CTGGTGCGCCACTACTAACA
SW15073	7RS	ATCCGTTCGCGTATCAAATG	CATACAAACGTGGATGCTGC
SW15541	7RS	TGGTTGATTTATCGGCCATT	CTTGCACTCCCTCATCCAAT
SW16052	7RS	CTTGAGGCTGGTGATAAGGC	ATTGGAGACTTGCTTGTGCC
SW16324	7RS	GTGTCACATCATGGCGTAGC	TGCTACAACGGGTCACAAAC
SW16916	7RS	ATGAGGCAGTCAGGCAAGTT	CACTGTAGCCACGCCCTTAT
SW17205	7RS	CCATTCTATTATTGCCTCTATGCC	AAGGAGCCAAGCTAGCAACA

SW17336	7RS	TTTCCTATCCCGACGATCAG	CTGTCACAGTTTACAGGGCG
SW21292	7RS	GTTGGGCCTTCTGGGTATCT	CGACGTCCATCACATCATTC
SW25893	7RS	CCCAGCTTCATCGACCTAGA	TGTTCCGCATTGTGAATGTT
SW26402	7RS	GTGCATAGTCTGGGCAACAG	TCTGCTCACTATCACACACGG
SW31197	7RS	CATTCCGATGTCCAAACTGA	AGGAGATGGTGCTGGTGAAG
SW31799	7RS	AAGATCAATTTCCTCTGGACACA	TAGCCGTGGTAGATGCAGTG
SW22055	7RS	ACCAAACCAAACCAAACCAA	CGCATATGGCAGAGAAGAAA
SW22990	7RS	GGATACCTGGTTTGTCTGCC	GAAACAGAACAGCGCAGGAT
SW36004	7RS	TGGAATAGTTGTTTGCACTGTTG	CGCTATTGGACCAGAAAGGA
SW49799	7RS	AACAAACAGGATCAGCACCC	TTGAGGGTGAACATGTGGAA
SW52977	7RS	AGGTCGATGATGTAGGCGAG	AGATCTGAGTTGGGCTCCTG
SW63468	7RS	ATCGTGGAACTTCAACCACA	TCTTAATACAGGTGCATTGGTCA
SW655348	7RS	GCGCTACTGTTAAGCCTCCA	TGAAACCACGATCAAACATGA
SW74731	7RS	GCGCTTCTGTCTGTGCTGTA	TCATTGCAGGCAATAATCCA
SW79635	7RS	AGCGAATCCGCTGAAGATAA	AATCGATCGACGAAATTGCT
SW852761	7RS	TCGGCTTGGAATCTTTCTCA	TTGATTCGGTCACGTCAGTC
SW33563	7RS	GAAGAATGCTTTCCCACACC	ACGTTGGAGACGTATTAGGGT
SW35194	7RS	GTCTAAGCTGACGGCTGTGG	CTTCGGAGTCTCGGAGCTT
SW13002	7RL	TTTAGAATCTCATGGCCGGT	TGACACGTGGCTTTCTTGAG
SW13721	7RL	CTCTCCCTCGTGCTCTCATC	GGCTCTGTCATTGTTGCTCA
SW14641	7RL	TATCAGGGTTGTCTCCCTGG	TGCTTAACACTTAGCGGCATT
SW15135	7RL	TTTCCAATGGACTATAACATACGG	CAATTGCAGTAGCAGTTGCG
SW16077	7RL	GCTGCATGGGAGCTAGATTT	GCAAATAATGCTAAACATCCGA
SW16524	7RL	CTCATTCGCTGACATCTGGA	CGGAGGGTTTAGTAGGCTAGG
SW168844	7RL	CCACAAGTCAGCCTCCAAAT	ATCGAATCTTCCTCAGCTCG
SW17001	7RL	TCCTACGGTATCCGGGAGTT	TGGACACCTTCAACAACGAC
SW19865	7RL	AAGAAATGAAGTTGCAGTGTCG	ATGCAACTCCAAACCTGTCC
SW21762	7RL	TTGTCCTCATCCTTCCGAGT	TGAAATGCATTGAGCAAAGG
SW22082	7RL	GCACTCTTACTTGCTCGTTGC	GGCATGTTGCAGTCACTTGT
SW22162	7RL	GCCACTAAGCATACACACATGG	GATGACGTGGCTAGAGAGGG
SW22257	7RL	TTTCCTCATCCGTTAGGCTG	ACCTTGGTCTCATCACCGAG
SW24787	7RL	GAGATTGTTCCTCGAATGCC	TCTAAACCTAGCCACCCAGC
SW25556	7RL	TTTCCCAACAATTTAAGCCC	TCAGATGCATAATTTCTTTCGG
SW25988	7RL	AAGGTCAAATGACCCGTGAA	CGTGACAACAACACATTACACC
SW28772	7RL	GTTCGATGAAGAGCATGGGT	AGAAGCCACTTGGGAGAACA
SW32263	7RL	TAGATATCAGCCGCCTCCTG	AATGAGTCACCGAGCCAGAC
SW28767	7RL	TATAGACCTCGCGTGTCACC	AGCAAGACGATTGCTTGATG
SW39642	7RL	TGCATCGATTCAGCACCTAC	CAACGATCTCACAGTGCTCC
SW41995	7RL	ACTTCAGGCGCTACATGGAC	CGCAAAGGACCCTTGTAATC
SW4741	7RL	CCCATTTGACCACATAATCCA	TCGTCCGGTGGGAATATTTA
SW61930	7RL	TCCCGGCCTAGGATTTAGTT	TCATCGGAGCGAACTGTATG
SW6485	7RL	CCATGTGACATTTGGTTTATGG	TCCTACGGTATCCGGGAGTT
SW8327	7RL	TTGAGAGTAGCCTTACCGGC	CAGTGCAACCAACATCGTTC
SW10134	7RL	TGCTTTCTACGCAACACACG	CATCGGTTCCACTCTTGGTT

SW21692	7RL	CCCTAATTGAGCATCTAGGGAA	ACCAGACCGTGTGGAGGA
SW233830	7RL	TGCTGCATTCTCTCAGCATT	GAGGTCCGTTCCTCTCTTCC
SW35950	7RL	GCACTGATGCTGTTGCAAAT	CTAAACCTGCGGTGGTGAAT
SW53066	7RL	GCAGTGATGGTGGAAGGACT	GCATTAACACCCGATAAGCG
SW54417	7RL	AGATCACAGCACAAGCTCGC	GCTATCCTTTACACCGCCAA
SW55626	7RL	GCTCGATCGCCTGAACTATC	CATGGGAATATTGCAGGACC
SW64011	7RL	AGCTCCATTGATGCTGGATT	GACAATAGAGCCTTGCTGCC
SW752962	7RL	CTTTGTCATGATGGTTTGGG	CCTGCAATTTCATGTGTTCG
SW86543	7RL	CTGGGCATGTTGTTGGTTTA	ACATACTTCGCTGAATGCCC
SW87447	7RL	TTGCATGTGGATCATGGTAA	ATCGAGGACAAAGGAACGG
SW94628	7RL	TCATTGTCAGTGGACCAACG	AACAACTGGAAGGCCTGATG
