# Supplementary material for: Scale development and utilization of universal PCR-based and high-throughput KASP markers specific for chromosome arms of rye (Secale cereale L.)
Source: BMC Genomics. 2020 Mar 4;21:206. doi: 10.1186/s12864-020-6624-y (PMC7057559; doi:10.1186/s12864-020-6624-y)
Supplement: Supplementary file 2 — Additional file 2: Table S2. Detection of 1RS translocations in 161 wheat cultivars/ lines. [file 12864_2020_6624_MOESM2_ESM.pdf]

Table S2 Detection of 1RS translocations in 161 wheat cultivars/ lines

No.	Cultivars/Lines	Origin	1RS translocations	No.	Cultivars/Lines	Origin	1RS translocations
1	Lumai5	Shandong	P	82	Shijiazhuang8	Hebei	P
2	Lumai13	Shandong	N	83	Hengguan35	Hebei	P
3	Lumai23	Shandong	N	84	Jinfeng6164	Hebei	P
4	Liangxing66	Shandong	N	85	Hanzao1	Hebei	P
5	Liangxing99	Shandong	N	86	Hanmai9	Hebei	P
6	Jimai19	Shandong	N	87	Hanmai13	Hebei	P
7	Jimai20	Shandong	N	88	Xingmai13	Hebei	P
8	Jimai22	Shandong	N	89	Ji518	Hebei	P
9	Jinan17	Shandong	N	90	Jimai585	Hebei	P
10	Tainong18	Shandong	N	91	Jishi02-1	Hebei	N
11	Tainong19	Shandong	N	92	Shiluan08-4	Hebei	P
12	Jinan16	Shandong	P	93	Cunmai1	Henan	P
13	Shannong17	Shandong	N	94	Zhengmai9023	Henan	N
14	Luyuan502	Shandong	P	95	Zhengmai9405	Henan	P
15	Shannong21	Shandong	N	96	Zhongyu9398	Henan	N
16	DH5197	Shandong	P	97	Zhongyuan6	Henan	P
17	Wennong14	Shandong	N	98	Zhoumai32	Henan	P
18	Lumai21	Shandong	N	99	Xuke718	Henan	P
19	Yannong19	Shandong	N	100	Zhoumai22	Henan	P
20	Yannong21	Shandong	N	101	Huaichuan916	Henan	N
21	Yannong23	Shandong	N	102	Zhoumai27	Henan	P
22	Weimai8	Shandong	P	103	Zhengzhou17	Henan	N
23	Yannong24	Shandong	N	104	Zhoumai9	Henan	P
24	Jimai21	Shandong	N	105	Zhoumai11	Henan	P
25	Lumai15	Shandong	P	106	Zhoumai13	Henan	P
26	Lumai14	Shandong	P	107	Zhoumai16	Henan	P
27	Lumai11	Shandong	P	108	Zhoumai17	Henan	P
28	Taishan9818	Shandong	P	109	Zhoumai18	Henan	P
29	Jinan18	Shandong	P	110	Zhoumai19	Henan	N
30	Dekang961	Shandong	N	111	Yumai13	Henan	P
31	Jinan20	Shandong	N	112	Wenmai8	Henan	P
32	Linmai2	Shandong	N	113	Zhengyou7	Henan	P
33	Linmai4	Shandong	N	114	Zhengnong16	Henan	N
34	Jining16	Shandong	N	115	Yumai47	Henan	N
35	Jining17	Shandong	P	116	Yumai29	Henan	N
36	Jining18	Shandong	P	117	Bainong207	Henan	N
37	Zimai12	Shandong	N	118	Aikang58	Henan	P
38	Zimai17	Shandong	N	119	Xinmai13	Henan	N
39	Shannong16	Shandong	N	120	Zhou8425B	Henan	P
40	Shannong15	Shandong	N	121	Yumai17	Henan	P
41	Jinghe9123	Hebei	P	122	Yumai18	Henan	N

42	Gao8901	Hebei	N	123	Yumai21	Henan	P
43	Gaoyou503	Hebei	N	124	Yumai68	Henan	P
44	Gaoyou9407	Hebei	N	125	Zhengyin4	Henan	N
45	Gaoyou9409	Hebei	N	126	Xinmai18	Henan	N
46	Gaoyou9415	Hebei	N	127	Bainong64	Henan	N
47	Cang6002	Hebei	N	128	Wenmai6	Henan	N
48	Cang6005	Hebei	N	129	Zhengmai366	Henan	N
49	Shixin5071	Hebei	N	130	Zhongyu8	Henan	P
50	Shixin633	Hebei	N	131	Xinmai11	Henan	N
51	Shixin733	Hebei	N	132	Shan225	Shannxi	N
52	Shixin616	Hebei	P	133	Shan229	Shannxi	P
53	Shixin828	Hebei	N	134	Xinong889	Shannxi	N
54	Shi4185	Hebei	P	135	Xinong979	Shannxi	N
55	Jiemai19	Hebei	N	136	Xinong881	Shannxi	N
56	Ji5265	Hebei	P	137	Xinong2611	Shannxi	N
57	Jimai11	Hebei	N	138	Xinong1718	Shannxi	P
58	Jimai36	Hebei	P	139	Xinong9718	Shannxi	N
59	Jimai38	Hebei	N	140	Xinong2208	Shannxi	P
60	Xiaoyan41	Hebei	N	141	Ligao6	Shannxi	N
61	Xiaoyan60	Hebei	N	142	Shan253	Shannxi	N
62	Hanyou1	Hebei	P	143	Changwu131	Shannxi	N
63	Shiyou17	Hebei	N	144	Changwu134	Shannxi	N
64	Shiyou20	Hebei	N	145	Xinong928	Shannxi	N
65	Shimai15	Hebei	P	146	Xinong2611	Shannxi	N
66	Shimai18	Hebei	P	147	Xiaoyan343	Shannxi	P
67	Shimai22	Hebei	P	148	Xiaoyan597	Shannxi	P
68	Kenong1006	Hebei	P	149	Xiaoyan246	Shannxi	N
69	Kenong1008	Hebei	P	150	Shanmai150	Shannxi	N
70	Kenong199	Hebei	P	151	Shan354	Shannxi	P
71	Kenong9204	Hebei	P	152	Xiaoyan54	Shannxi	N
72	Heng4422	Hebei	P	153	Xuzhou24	Jiangsu	N
73	Heng4444	Hebei	P	154	Xuzhou25	Jiangsu	N
74	Heng4399	Hebei	P	155	Xuzhou26	Jiangsu	P
75	Heng5471	Hebei	P	156	Xuzhou27	Jiangsu	N
76	Henong826	Hebei	P	157	Xuzhou856	Jiangsu	P
77	Henong827	Hebei	P	158	Huaimai18	Jiangsu	P
78	Han4589	Hebei	P	159	Huaimai20	Jiangsu	P
79	Han5316	Hebei	P	160	Wanmai46	Anhui	P
80	Han6172	Hebei	P	161	Wanmai38	Anhui	N
81	Han7086	Hebei	P				

P indicates presence of 1RS translocations; N indicates absence of 1RS translocations.
